# ﻿Species of *Peperomia* (Piperaceae) from the Saña River Valley, Peru

**DOI:** 10.3897/phytokeys.225.99277

**Published:** 2023-04-19

**Authors:** Guillermo Eloy Pino Infante, Marie-Stéphanie Samain, Joaquina Adelaida Albán Castillo, Luis Enrique Aarón Alomía Collazos

**Affiliations:** 1 Universidad Nacional Mayor de San Marcos, Museo de Historia Natural, Av. Arenales 1256, Jesús María, Lima 15072, Peru Sociedad Peruana de Cactáceas y Suculentas Lima Peru; 2 Sociedad Peruana de Cactáceas y Suculentas, Av. 6 de Agosto 1146, Jesús María, Lima 15072, Peru Universidad Nacional Mayor de San Marcos Lima Peru; 3 Instituto de Ecología, A.C., Centro Regional del Bajío, Red de Diversidad Biológica del Occidente Mexicano, Av. Lázaro Cárdenas 253, 61600 Pátzcuaro, Michoacán, Mexico Instituto de Ecología, A.C., Centro Regional del Bajío, Red de Diversidad Biológica del Occidente Mexicano Pátzcuaro Mexico

**Keywords:** Amotape-Huancabamba Zone, Andean cloud forest, Cajamarca, *Fenestratae*, Lambayeque, *Panicularia*, western slopes of the Andes

## Abstract

The Saña River Valley in Northern Peru is unusual for the western slopes of the Peruvian Andes because of its nearly year-round regime of precipitation instead of marked seasonal dry winters. This results in unexpected plant diversity. We surveyed the species of *Peperomia* (Piperaceae), occurring in this valley from 300 to 3000 m elevation, based on the study of specimens from ten herbaria and field collections, resulting in a total of 81 accessions, of which 48 were made by the authors. We found 16 different taxa: *Peperomiacacaophila*, from Ecuador, is reported for the first time in Peru; *P.cymbifolia*, *P.dolabriformis* and *P.emarginulata* are reported for the first time for the Saña River Valley; other widely distributed species like *P.fraseri*, *P.galioides*, *P.haematolepis*, *P.hispidula*, *P.inaequalifolia*, *P.microphylla*, and *P.rotundata* were also found. Five species new to science are described: *P.pilocarpa*, *P.riosaniensis*, close to *P.palmiformis* from Amazonas; *P.sagasteguii*, related to *P.trinervis*, *P.symmankii*, close to *P.ricardofernandezii* from Piura, and *P.vivipara*, related to *P.alata.* A key to the species of *Peperomia* from the Saña River Valley, based on vegetative characters, is provided.

## Abstract (es)

El valle del Río Saña en el norte de Perú es inusual para las vertientes occidentales de los Andes peruanos por su régimen de precipitación ininterrumpido en lugar de inviernos secos estacionales marcados. Esto resulta en una diversidad de plantas inesperada. Revisamos las especies de *Peperomia* (Piperaceae), del valle del Río Saña entre los 300 a 3000 m de altitud, basándonos en el estudio de especímenes de diez herbarios y colecciones de campo, resultando en un total de 81 accesiones, de las cuales 48 fueron realizadas por los autores. Encontramos 16 taxones diferentes: *Peperomiacacaophila*, de Ecuador, se reporta por primera vez en Perú; *P.cymbifolia*, *P.dolabriformis* y *P.emarginulata* se reportan por primera vez para el valle del Río Saña; otras especies ampliamente distribuidas como *P.fraseri*, *P.galioides*, *P.haematolepis*, *P.hispidula*, *P.inaequalifolia*, *P.microphylla* y *P.rotundata* también se encontraron. Se describen cinco especies nuevas para la ciencia: *P.pilocarpa*, *P.riosaniensis*, cercana de *P.palmiformis* de Amazonas; *P.sagasteguii*, relacionada con *P.trinervis*, *P.symmankii*, cercana a *P.ricardofernandezii* de Piura, y *P.vivipara*, relacionada con *P.alata.* Se presenta una clave para las especies de *Peperomia* del Valle del Río Saña basada en caracteres vegetativos.

## ﻿Introduction

*Peperomia* (Ruiz & Pavón, 1794), was the name chosen to distinguish some species previously described by Linnaeus as *Piper*, and its first species were described in Peru (Ruiz and Pavón 1798). It is the second largest genus of the family Piperaceae after *Piper*, with a pantropical distribution of about 1,600 species (Samain et al. 2009; Mathieu et al. 2011; Samain et al. 2011). Most of them occur in the Neotropics, and approximately 400 species can be found in Peru (Trelease 1936). Due to its high species number, subgenera were proposed and used for classification, based only on morphology (Miquel 1843; Dahlstedt 1900) until this century, when their phylogeny was clarified using molecular studies (Wanke et al. 2006; Samain et al. 2007; Samain et al. 2009). Finally, Frenzke et al. (2015) published a new infrageneric classification as a reference for studies in the genus, based on fruit characters, macroscopic features, and molecular data, defining 14 monophyletic groups, and describing 5 new subgenera. Peru hosts representatives of all subgenera (Frenzke et al. 2015), and it also has the highest diversity of species in the world (Ulloa et al. 2017), many of which are still not described, whereas a considerable number of the currently accepted species are possible synonyms (unpublished results of the first author).

While some of the subgenera are highly diverse and widely distributed (e.g., *Micropiper* (Miq.) Miq., *Leptorhynchum* (Dahlst.) Trel. ex Samain, *Pseudocupula* Frenzke & Scheiris), others are small and restricted to the Neotropics, and even to some Andean countries (Colombia, Ecuador, Peru, and Bolivia) (e.g., *Perlucida* Scheiris & Frenzke, *Phyllobryon* (Miq.) Scheiris & Frenzke). Among these small groups, two of them were split from the former subgenus Panicularia sensu Dahlstedt, into two well-supported clades that are well characterized by morphological features. (*Panicularia* Miq. and *Fenestratae* Pino) with most of their species adapted to seasonal dry periods through succulence and circumscribed to Peru and Ecuador (Pino et al. 2020). Due to the lack of information, many species of these two groups need to be reviewed and new species are to be described. In the quest for these novelties, we explored the Saña River Valley, in the north of Peru, where we found many taxa together in a small area, that was not known to be diverse for *Peperomia*. Therefore, the objective of the present paper is to review the species of *Peperomia* present in the Saña River Valley, Peru, as a sample of the high diversity of this species existing in the country.

## ﻿Materials and methods

### ﻿Study area

The Saña (also spelled Zaña) River is a short course (ca. 125 km length) river on the western slopes of the northern Peruvian Andes, with two main tributaries: Udima River and Nanchoc River. Altogether they make up a total drainage area of 1754.7 m^2^ (ANA 2010). The river rises in the district Calquis, province San Miguel, department Cajamarca, at approximately 3200 m elevation (ANA 1999), notoriously lower than similar rivers draining the Pacific Ocean basin.

**Figure 1.**
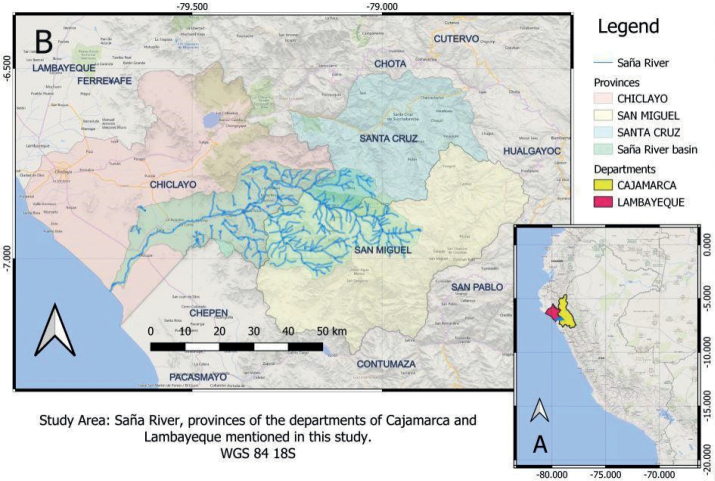
Study area **A** map of Peru and the localization of the departments Lambayeque and Cajamarca **B** close-up to show the Saña River and its basin (light green) shared by the provinces of Chiclayo (Lambayeque), San Miguel, and Santa Cruz (Cajamarca).

After crossing the districts of Calquis and La Florida in the province of San Miguel and the district of Catache in the province of Santa Cruz (both provinces of the Cajamarca department), it enters the Oyotún district of the province of Chiclayo, Lambayeque department, after which it crosses the districts Nueva Arica, Cayaltí, Zaña, and Lagunas, as it flows to the sea across the coastal desert of the southernmost part of the Lambayeque department, close to the border with the department of La Libertad (Fig. 1).

Unlike the Chancay-Reque River system located just north and the Jequetepeque River Valley South of it–both with typical coastal desertic valleys–the Saña River Valley is more humid on average, due to the influence of the Amotape-Huancabamba Zone (AHZ) (observation of the first author). This zone has been proposed as a biodiversity hotspot for its higher diversity levels more than adjacent zones (Weigend 2002). Its boundaries have been set from Río Jubones and Río Zamora systems in Ecuador to the Río Chicama system in Peru (La Libertad). Thus, its influence reaches the highlands of Lambayeque and particularly the Saña River Valley, where the humid forests on the western slopes of the Andes can only be explained by the relatively low elevation of the Andes in the AHZ, which allows the flow of moisture from the rainforests of the Amazon Basin on the east to the desert coasts of western Peru. Plants and animals typical of the Amazon Forest e.g. military macaws (*Aramilitaris* L.) were reported here at the highest altitudes of the Saña River Valley (Taulis) before the anthropogenic deforestation of recent decades, (Koepcke 1961). Native forests of its middle elevations have been displaced by cultivated patches of the tropical bamboo *Guaduaangustifolia* Kunth (Caña Guayaquil) which is extensively cultivated in northern Peru, but not in the adjacent valleys.

From the coast to the Andes, close to the riverbanks of the Saña River, from west to east, we find the localities of Lagunas, Mocupe, Zaña, Cayaltí, La Viña, Culpón, and Nueva Arica. After a detour of the road to the town of Nanchoc in the South, the river flows northwards to the town of Oyotún and Pan de Azúcar, turning later southeastward to El Espinal and La Florida, where a detour to the South moves the road away from the river to El Naranjo and Niepos. Beyond Nanchoc, there is a small relict forest called “La Oscurana” after the town of Bolívar, with four species of *Peperomia* reported, although only one was found in the Herbaria (Juárez et al. 2005). From La Florida, another small road continues east and then southwards along the Saña River leading to the former Taulis Estate, now a hamlet and no longer a cloud forest due to intense cultivation. From El Espinal on a short walk northwards, we find the Velo de Novia waterfall. Before reaching La Florida, after the bridge El Papayo over the Saña River, a detour to the northeast leads to the town of Monteseco, a former estate located south of a relict forest. From here, a trail leads to El Chorro Blanco waterfall and the former Udima Estate crossing the forest. River Udima, a northeastern affluent of Saña River, crosses the forest from east to west and flows into it at El Espinal.

The fauna and flora of the remaining forest North of Monteseco have been published by Cadle (1989), Cadle and McDiarmid (1990), and Sagástegui and Dillon (1991). The latter report 88 families, 200 genera, and over 326 species of ferns and flowering plants, among them nine species of *Peperomia*, in a cloud forest where native trees are covered with mosses, ferns, orchids and vines, and humidity is maintained by multiple streams and a waterfall. The forest has been partially destroyed by logging that occurred until the 1960s, and only patches of natural vegetation are left (Sagástegui and Dillon 1991). The Peruvian government has protected this area since 2010, declaring it as a reserved zone: “Zona Reservada Udima” (El Peruano 2010), and categorizing it as “Zona Reservada Udima en Refugio de Vida Silvestre, Bosques Nublados de Udima” (El Peruano 2011).

From 30 to 500 m elevations, the warm desertic climate with temperatures of up to 35 °C in summer (Clima.com) favors the abundance of cactus steppes intermingled with densely irrigated rice and sugar cane cultivation that almost dry up the river at this altitude. From 500 m upwards, the climate changes abruptly to temperate and humid with an annual average temperature of 18 °C. Most cacti grow below 500 m, but some species reach 1000 m of elevation. The unexpected diversity of species of cacti in such a coastal valley was described first by Mischler (1988) and more recently by Alomía (2020), who mentions 13 taxa: six are endemic to Peru but only the last one in the following list is endemic to the Saña River Valley; *Armatocereusoligogonus* Rauh & Backeb., *Espostoamelanostele* (Vaupel) Borg, *Haageocereusicosagonoides* Rauh & Backeb., *Haageocereuspseudoversicolor* Rauh & Backeb., *Neoraimondiaarequipensis* (Backeb.) Buxb. and RauhocereusriosaniensisBackeb.ssp.riosaniensis. Other plants adapted to this xerophytic environment are *Burseragraveolens* (Kunth) Triana & Planch, *Mimosapellita* Kunth. ex Willd, *Rauvolfiatetraphylla* L., Vachelliaaromavar.huarango (Ruiz ex J.F.Macbr.) Seigler & Ebinger and *Vallesiaglabra* Link. (Sagástegui and Dillon 1991). From this altitude up to 2500 m, as precipitation increases, dense forests are seen, a combination of dry western Andean forests and eastern montane forests (observation by the first author). During the summer months (December to May), increased rain can cause downstream floods, that occasionally reach the lower basin, such as the one that wiped out the city of Zaña in 1720 (Angulo 2011).

Although the abovementioned studies have identified nine species of *Peperomia* in the relict humid forest north of the town of Monteseco and four species of *Peperomia* in the forest of La Oscurana, so far no research has been carried out for the rest of the valley, especially the drier areas where fewer species are supposed to occur. This study aims to assess the diversity of *Peperomia* species along the whole Saña River valley, adding new reports of already known species and describing new ones.

### ﻿Plant material

This research was approved by the Bioethics Committee of the Facultad de Ciencias Biológicas de la Universidad Nacional Mayor de San Marcos, document 009-2021-CBE-FCB-UNMSM. Collections were enabled by the Servicio Nacional Forestal y de Fauna Silvestre (SERFOR) permit numbers 009-2009-AG-DGFFS-DGEFFS, 0124-2011-AG-DGFFS-DGEFFS and authorization n° AUT-IFL-2018-064 issued by RDG N° 491-2018-MINAGRI-SERFOR-DGGSPFFS extended by RD N° D000170-2021-MIDAGRI-SERFOR-DGGSPFFS-DGSPF. Fieldwork was carried out by the teams of the Ghent University Botanical Garden (Belgium) and the Technische Universität Dresden (Germany) in 2009, and later by the Museo de Historia Natural – Universidad Nacional Mayor de San Marcos in the years 2020–2022, along the Saña River Valley starting from Zaña city up to Niepos, including the departments of Lambayeque (prov. Chiclayo) and Cajamarca (prov. San Miguel, La Florida, and Santa Cruz). No collections were made in the protected zone of Udima.

Field observations were complemented with the study of herbarium specimens of ten herbaria: F, GB, HAO, HLL, HUA, HUT, NY, UC, US, and USM, checking the types online. (acronyms following Thiers 2022). A total of 81 collections were included, of which 48 were made by the authors.

Photographs with either Sony DSC-HX400V or Olympus TG-6 cameras were taken of plants in habitat and plants *ex-situ* (vegetative, reproductive, and young plants). Flowers and seeds were photographed either with macro lenses, OPPO Reno-7, or stereomicroscopes. Measurements were taken from fresh plants, photographs, and dry herbarium specimens. Whenever possible, three or more measurements per structure. Floral organ measurements were based on photographs using ImageJ 1.53t. Colors were based on photographs of living plants and notes on herbarium labels.

All localities were georeferenced using GPS coordinates (https://www.gps-coordinates.net/) and Google Maps. For herbarium specimens without coordinates, approximate coordinates were calculated using Google Earth. Maps of distribution were drawn using the same set of coordinates using QGIS v.3.26 Buenos Aires (Official website QGIS: https://www.qgis.org/en/site/).

Finally, the subgenus to which each species belongs is mentioned according to the infrageneric classification of the genus *Peperomia* by Frenzke et al. (2015), who assigned all species described until then to their respective subgenus based on morphological and/or molecular data. The subgeneric placement of the new species described in this paper was defined based on morphological characters.

## ﻿Results

### ﻿Taxonomy

**Figure 2.**
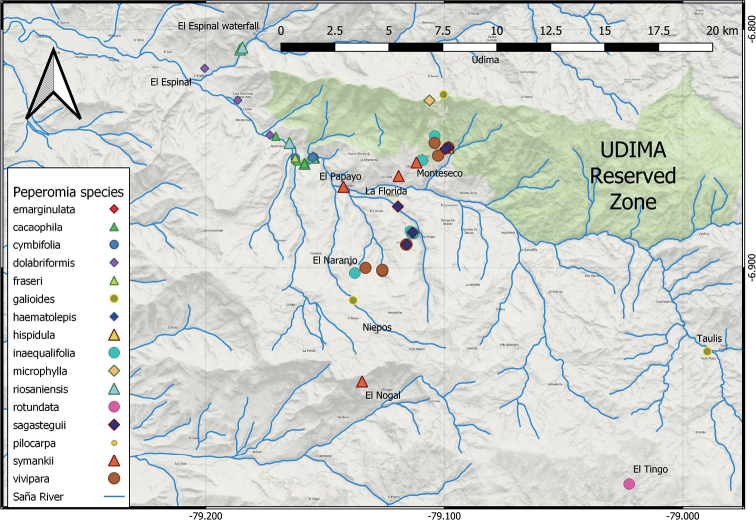
Distribution of the *Peperomia* species in the study area. Udima reserved zone in green shadow.

The present study found 81 accessions of 16 different taxa of *Peperomia* in the Saña River Valley (Fig. 2). We here present an identification key for these taxa. *Peperomiacrystallina*, *P.hispiduliformis*, and *P.hartwegiana* are included in the key due to the high probability of occurrence in the region. Characteristics of the species were taken from the present study as well as Pino (2004), Scheiris (2013), Steyermark (1984), and Trelease and Yuncker (1950).

### ﻿Key to the species of *Peperomia* from the Saña River Valley

**Table:** 

1	Leaves papyraceous, stems friable, hispid, and transparent	**2**
–	Leaves succulent or semi-succulent, stems firm, opaque	**4**
2	Stems erect, trichotomously branched, spadices in zigzag-shape, seemingly sessile on leaves, fruits verrucose	***Peperomiacrystallina* Ruiz & Pav.**
–	Stems shortly erect, then subdecumbent, spadices straight, originating from upper leaf axils, hispid fruits	**3**
3	Branches inserted at less than 45°, leaves ovate-orbicular, apex and base obtuse, venation pinnate	***Peperomiahispidula* (Sw.) A. Dietr.**
–	Branches inserted at 45–90°, leaves cordiform, apex round to obtuse, base cordate, venation palmate	***Peperomiahispiduliformis* Trel.**
4	Lower leaves in basal rosette, cordate, only reproductive stem growing upwards	***Peperomiafraseri* C. DC.**
–	Lower leaves not in a basal rosette, leaves growing along a long stem	**5**
5	Leaves alternate	**6**
–	Leaves opposite or whorled	**14**
6	Leaves vertically compressed, very succulent, adaxial size reduced, epidermis covering the internal hydrenchyma, and exposing the photosynthetic chlorenchymatous tissues to solar irradiance through the abaxial surface either directly or by reflection from the ground. (fenestra), inflorescence a panicle	**7**
–	Leaves not vertically compressed, adaxial size similar to abaxial side in size, succulent, semi-succulent or chartaceous	**8**
7	Leaves oval elliptic, papillated surface, gray-glaucous to reddish dull green	***Peperomiacymbifolia* Pino.**
–	Leaves dolabriform, smooth surface, light green to glaucous	***Peperomiadolabriformis* Kunth.**
8	Leaves succulent, spirally crowded at the distal end, stem erect, succulent, inflorescence a panicle	***Peperomiariosaniensis* Hutchison ex Pino, Samain & L.E. Alomía, sp. nov.**
–	Leaves semi-succulent or chartaceous, all along the stem, inflorescence simple, geminated or loosely flowered	**9**
9	Stems many-branched distally and horizontally, plants densely pubescent	***Peperomiasagasteguii* Pino, Samain & L.E. Alomía, sp. nov.**
–	Stems branching basally and at different angles, glabrous or puberulous	**10**
10	Leaves distichous, stem winged	***Peperomiavivipara* Pino, Samain & L.E. Alomía, sp. nov.**
–	Leaves spirally attached	**11**
–	Leaves opposite or whorled	**15**
11	Leaf base cordate, subpeltate, stem succulent, tortuous, inflorescence a panicle of clusters of spadices	***Peperomiasymmankii* Pino & Samain, sp. nov.**
–	Leaf base cuneate, lamina elliptical to ovate or obovate, seeds sticky	**12**
12	Leaves with round, truncate, or emarginate apex	***Peperomiahaematolepis* Trel.**
–	Leaves with acute, obtuse to attenuate apex	**13**
13	Stems erect, short, quadrangular in section, leaves elliptic-obovate, spadices white, terminal in umbels of 3, whitish, 8–12 cm long	***Peperomiaemarginulata* C. DC.**
–	Stems trailing, long, terete, leaves elliptic ovate or obovate, spadix terminal simple, reddish or reddish green	**14**
14	Stems without wings, leaves ovate, acute to acuminate, succulent, canaliculated	***Peperomiacacaophila* Trel. & Yunck.**
–	Stems with two small wings, leaves widely obovate,, apex obtuse, semisucculent, flat	***Peperomiapilocarpa* Pino, Samain & L.E. Alomía, sp. nov.**
15	Leaves chartaceous, opposite or decussate, strongly pubescent leaves and stems	***Peperomiarotundata* Kunth**
–	Leaves succulent, verticillate, glabrous to puberulous	**16**
16	Leaves orbicular to oval, inflorescence terminal simple, red	***Peperomiahartwegiana* Miq.**
–	Leaves narrow elliptical to obovate, inflorescences yellow or green	**17**
17	Whorls of 4–5 leaves, small (usually less than 1 cm long), all homogeneous in size and thickness, inflorescence simple, terminal	***Peperomiamicrophylla* Kunth**
–	Whorls of 6–8 leaves, juvenile and mature leaves of different size and thickness, inflorescences in terminal umbellas and axillary from distal nodes	**18**
18	Plants large (25–50 cm tall), stems 1.2–4.5 mm diam., basal leaves 6–12 mm long, terrestrial or lithophytic, above 2200 m	***Peperomiagalioides* Kunth**
–	Plants small (10–24 cm tall), stems 3.5–8 mm diam., basal leaves 4–7 mm long, epiphytical, below 2000 m	***Peperomiainaequalifolia* R. & P.**

#### ﻿1. Peperomia cacaophila

Taxon classificationPlantaePiperalesPiperaceae

Trel. & Yunck., Piperac. N. South Amer. 2(610). 1950.

5E24A57C-91B9-5BDC-845E-F94857CDD5BF

Fig. 3A–F

##### Type.

**Ecuador**, prov. Los Ríos, cant. Quevedo, roadside at Quevedo, common at Cacao groves, *Haught 2966* (holotype: US 1707621!, isotype: ILL).

**Figure 3.**
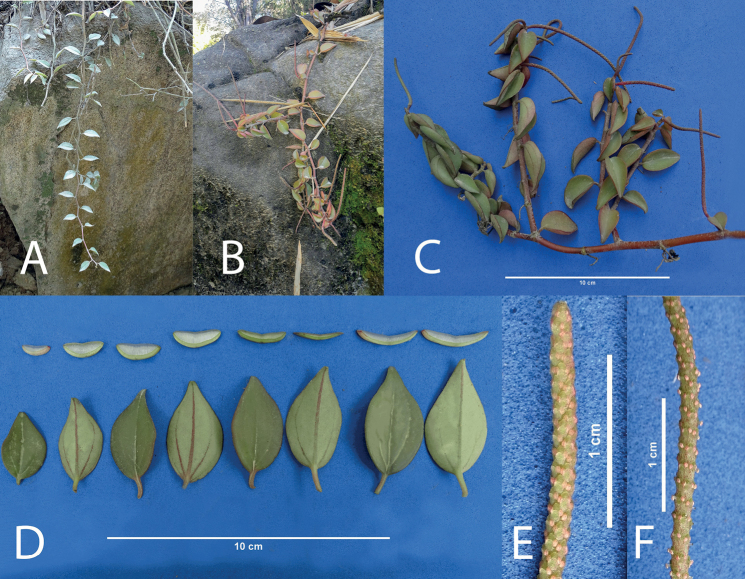
*Peperomiacacaophila* Trel. & Yunck **A** young plant hanging from a rock **B** long, trailing plant with distal inflorescence **C** plant *ex-situ* with several branches **D** detail of the leaves **E** spadix at the beginning of anthesis **F** mature spadix.

##### Distribution and habitat.

Plants were known only from central Ecuador, prov. Los Ríos, Guayas, Esmeralda, and Pichincha, epiphyte on *Theobroma* or similar trees, in shaded warm forests from 200–1500 m. This is the first report for Peru.

##### Notes.

The closest species we could compare to is *P.topoensis* Yunck., also from Ecuador, which is similar in its epiphytic, pendent, or assurgent habit with small, succulent, alternate ovate leaves, cordulate at the base and 7-plinerved, compared to the obtuse, subtruncate based and noticeable 3-nerved leaves of *P.cacaophila*. *Peperomianitida* Dahlst. from Brazil, São Paulo (Campinas), a widely cultivated species, is similar but with larger, light green cordulate-based leaves, only stems slightly reddish. *Peperomiaportobellensis* Beurl. from Panama is also similar in shape but with slightly larger leaves. No material other than the types of these species was available for further comparison.

This species belongs to Peperomiasubg.Micropiper (Miq.) Miq. (Frenzke et al. 2015).

##### Specimens examined.

**Peru, dept. Cajamarca, prov. Santa Cruz, dist. Catache**: Road to Hacienda Taulis, ca. 60 km east of Cayaltí on the Río Saña. 560 m, [06°50'41.5"S, 79°10'14.2"W], 28 Aug 1964, *P. C. Hutchison 6323* (UC, US, F 1642092, USM 16451); Road from El Espinal to La Florida, 550 m, 06°51'23.0"S, 79°09'31.0"W, Jul 26 2020, *G. Pino & L.E. Alomía 3234* (USM); Same road, 527 m, 06°51'16"S, 79°09'44"W, 28 Aug 2022, *G. Pino et al. 3625* (USM 330878); idem, 536 m, 06°51'18.6"S, 79°09'41.6"W, 28 Aug 2022, *G. Pino et al. 3630* (USM 330879).

#### ﻿2. Peperomia cymbifolia

Taxon classificationPlantaePiperalesPiperaceae

Pino, Pino, Peperomias de Cajamarca: 16–17, 50–51 f. 3 A–N. 2004.

C62106F4-BC8F-543B-8D63-D99883A59948

Fig. 4A–F

##### Type.

**PERU**, dept. Cajamarca, prov. San Pablo, dist. San Pablo, the road to Chilete, 2080 m, 14 May 1964, *P.C. Hutchison & J. K. Wright 5076* (holotype: UC 301913!, isotype: USM 16457!).

**Figure 4.**
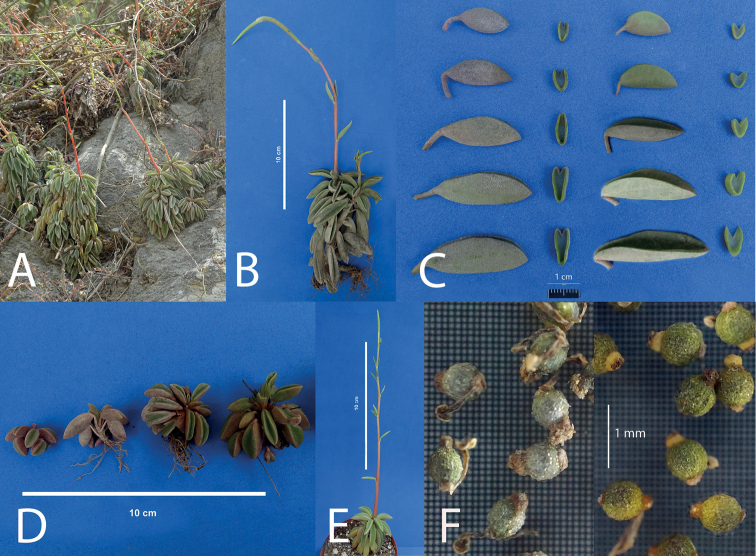
PeperomiacymbifoliaPinovar.occidentalis Pino & L.E. Alomía var. nov. **A** plant in habitat **B** plant *ex situ***C** comparison of the leaves of P.cymbifoliavar.cymbifolia (left) and var. occidentalis (right) **D** young plants showing development **E** cultivated plant showing inflorescence **F** seeds of P.cymbifoliavar.cymbifolia (right) and var. occidentalis (left).

##### Distribution and habitat.

Montane scrubs of the department of Cajamarca (Prov. San Pablo, Contumazá, Cajamarca, Chota, and San Marcos) between 1800 to 2800 m.

##### Notes.

The samples collected were determined as *P.cymbifolia* (Pino 2004) However, comparing the plants of the Saña River Valley and the plants from the type locality we found some differences summarized in Table 1. These differences do not support the description of a new species but are sufficient to describe a new variety of this species. Moreover, while previous collections grow from 1800 to 2800 m in colder and moister places, this variety seems to be adapted to low altitudes of 500 to 600 m with higher temperatures and drier periods. It is easy to cultivate outdoors in the city of Lima, quite different from var. cymbifolia, which requires a cool greenhouse. This species belongs to Peperomiasubg.Fenestratae Pino (Frenzke et al. 2015).

**Table 1.** Comparison of the main differences between P.cymbifoliavar.cymbifolia and var. occidentalis (Data from var. cymbifolia taken from Pino (2004), and from live plants).

Features	P.cymbifoliavar.cymbifolia	P.cymbifoliavar.occidentalis
Height (vegetative) in cm	10–20	5–15
Leaf (shape)	Adaxial side slightly canaliculate, Abaxial side convex.	Adaxial side strongly canaliculate, Abaxial side straight to slightly convex.
	Lateral faces flat to slightly convex or concave.	Lateral faces more constantly convex.
Leaf (length in cm)	(1.6–) 2.2–3.4	(1.4–) 1.9–4
Leaf (vertical height of lateral sides in mm)	9–13, flat to concave, nerves conspicuous	7–10, flat to convex, nerves obscure
Rachis of the spadix (length in cm)	(2–)3.8–6	7–15
Fruit (shape/color)	Globose, yellowish-green to brown	Ovoid to ellipsoid, light olive green
Fruit (length in mm)	0.7–0.8	0.6–0.8
Fruit (diam. in mm)	0.6–0.7	0.45–0.55
Fruit (stylopodium)	Broad conical, light brown,	Broad cylindrical, light brown – orangish,
	0.1–0.2 mm long,	0.2–0.25 mm long,
	0.2–0.3 mm diam.	0.2–0.35 mm diam.
Altitude range (m)	1800–2800	500–600

#### ﻿ Peperomia cymbifolia Pino var. occidentalis

Taxon classificationPlantaePiperalesPiperaceae

Pino & L.E.Alomía var. nov.

0F34B5DC-F472-558D-8EC5-434F7D2DA058

urn:lsid:ipni.org:names:77317651-1

##### Diagnosis.

Peperomiacymbifoliavar.occidentalis differs from the type variety mainly in size, plants are smaller and more compact; Leaves are slightly longer (4 cm maximum compared to 3.6 cm long), their lateral faces are not as high (7–10 mm compared to 9–13 mm), and they are more constantly convex than concave, its abaxial side is less convex, sometimes even straight; Its fruits are smaller and narrower, (0.6–0.8 × 0.45–0.55 mm compared to the 0.7–0.8 × 0.6–0.7 mm), light olive green instead of yellowish green, and with a relatively larger stylopodium. Populations of this variety are not only separated geographically from the type variety but mostly by altitude and climate: they grow 1200 m below the lowest occurrence of the type variety, at least 10 °Celsius warmer. The epithet reminds the localization of this variety, the most occidental of all three varieties of *P.cymbifolia*. (P.cymbifoliavar.cymbifolia ,var. goodspeedii , and var. occidentalis).

##### Type.

**Peru**, dept. Cajamarca, prov. Santa Cruz, dist. Catache: road from Espinal to La Florida, 521 m, 06°51'14.2"S, 79°09'45"W, 28 Aug 2022, *G. Pino & L.E. Alomía 3634* (holotype: USM 330881!).

##### Distribution and habitat.

Plants grow from 450 to 600 m, on exposed rock crevices facing southwards between the towns of El Espinal and La Florida in Department Cajamarca, province of Santa Cruz. A similar collection, very likely the same taxon, was found in Chota at 750 m, exactly 20 km to the North of the Saña River Valley in a straight line.

##### Phenology.

Inflorescences appear from June to February; fruits ripen from October to March.

##### Additional specimens examined.

**Peru, dept. Cajamarca, prov. Santa Cruz, dist. Catache**: Road from El Espinal to La Florida, 582 m, 06°51'13.7"S, 79°09'16.5"W, 26 Jul 2020, *G. Pino & L.E. Alomía 3232* (USM 330872); Same road, 591 m, 06°51'13.4"S, 79°09'18.4"W, 28 Aug 2022, *G. Pino et al. 3638* (USM 330883). **Prov. Chota, dist. Chimbán**, Road from Cumbil to Llama, [between Potrerillo and Maychil], border of the road, on rocks, 750 m, [06°31'56"S, 79°11'51"W], 21 May 1965, *A. López & A. Sagástegui s/n* (HUT 5520). **Dept. Lambayeque, prov. Ferreñafe, dist. Incahuasi**: Before Laquipampa, 721 m, 06°20'31.5"S, 79°26'41"W, 08 Mar 2023, *G. Pino & J.E. Romero 3840* (USM).

#### ﻿3. Peperomia dolabriformis

Taxon classificationPlantaePiperalesPiperaceae

Kunth, Nov. Gen & Sp. [Humboldt] 1: 50, tab. 4. 1815.

773CC97F-A032-5474-A50F-026A079E1419

Fig. 5A

Piper dolabriforme Poir., Lam. Encycl. Meth. Suppl. 4: 464. 1816. Type: Based on Peperomiadolabriformis Kunth.

##### Type.

**Peru**, dept. Piura, prov. Huancabamba: “in Peruviae calidis, ad ripas flumini Guancabamba” [in the warmth of Peru, on the banks of the Huancabamba River], *Humboldt s/n* (holotype: B [lost?], isotype P!).

**Figure 5.**
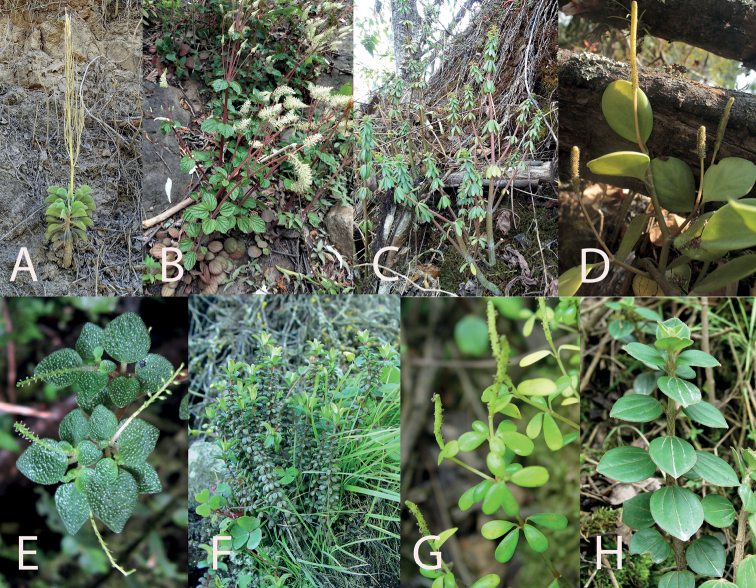
*Peperomia* species that can be found in the Saña River Valley **A***P.dolabriformis***B***P.fraseri***C***P.galioides***D***P.haematolepis***E***P.hispidula***F***P.inaequalifolia***G***P.microphylla***H***P.rotundata*.

##### Distribution and habitat.

Warm valleys of the Western slopes of the Andes of departments Lambayeque, La Libertad, and Cajamarca. Huancabamba, Marañón and Crisnejas Valleys in departments Piura, Cajamarca, Amazonas, La Libertad and Ancash (Sihuas) in Peru. In Ecuador, it occurs in the provinces of Loja and Zamora-Chinchipe.

##### Note.

This species belongs to Peperomiasubg.Fenestratae Pino (Frenzke et al. 2015).

##### Specimens examined.

**Peru, dept. Lambayeque, prov. Chiclayo, dist. Oyotún**: Canyon above Chiclayo near Mocupa [Mocupe, 391 m, 06°48'58.7"S, 79°12'01.6"W], Johnson Cactus Garden, prepared at the BG of the University of California at Berkeley, accession 52.498, 24 Sep 1958, *Larrabie s/n* (F1567768, NY, UC088167, US2301205) **Dept. Cajamarca, prov. Santa Cruz, dist. Catache**: road from Oyotún to El Espinal, 397 m, 06°49'46.7"S, 79°11'11.7"W, 28 Aug 2022, *G. Pino & L.E. Alomía 3613* (USM 330874); same road, 440 m, 06°50'39.9"S, 79°10'23.1"W, 28 Aug 2022, *G. Pino & L.E. Alomía 3621* (USM 330875).

#### ﻿4. Peperomia emarginulata

Taxon classificationPlantaePiperalesPiperaceae

C.DC. DC. Prodr. 185. 16(1): 433. 1869.

21A164DA-9FEA-5967-AF3B-47E2489030C4

Fig. 6A–F

##### Type.

**Peru**, dept. [Huánuco], Casapí, In Peruvia, sylvis Andium occident. [In Perú, western Andes forests], *Mathews 1687* (lectotype: K, designated by Mathieu and Callejas (2006: 351; isolectotype: E, K!)).

**Figure 6.**
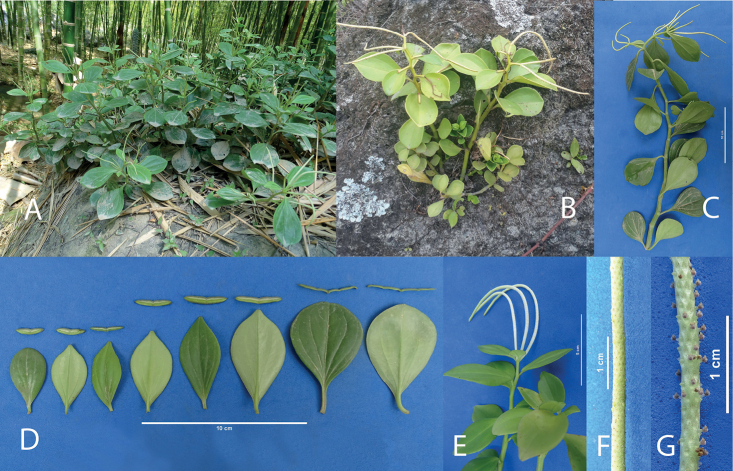
*Peperomiaemarginulata* C.DC **A** colony growing on fallen *Guaduaangustifolia* leaves **B** exposed plant growing on a rock **C** plant *ex-situ* showing 4-angled stems and inflorescences **D** detail of the leaves **E** terminal umbella with spadices **F** spadices at the beginning of anthesis **G** mature spadix with seeds.

##### Distribution and habitat.

Plants are reported from Ecuador and Peru, in montane forests from 300 to 1500 m.

##### Notes.

We compared the three species with features closer to our findings (Table 2) The plants we found are in the range of all three, but according to this table, it matches *P.emarginulata*. *Peperomiaangularis* and *Peperomiacaucana* from Colombia should be reviewed in the future to prove if they are conspecific with *P.emarginulata*. *Peperomiagarcia*-*barrigana* Trel. & Yunck. and *P.gabinetensis* Trel. & Yunck. also show similarities that deserve further analysis, being mostly different in size. Some specimens in herbaria were also determined as *P.glabella* (Sw.) A. Dietr., a widely distributed species, very close to this group, that is relatively small.

**Table 2.** Comparison of some features of the descriptions of *P.angularis*, *P.emarginulata*, and *P.caucana* with the specimens of *P.emarginulata* studied. All data according to Trelease and Yuncker (1950), complemented with Steyermark (1984) for *P.angularis* and Trelease (1936) for *P.emarginulata*.

Features	*P.emarginulata* of Saña River Valley	*P.angularis*	*P.emarginulata*	*P.caucana*
Height in cm	15–45	25 or more	30 or more	15 or more
Stem (diam. in mm)	3–5	1–3	4–5	2
Internodes in cm	1.5–4	1–3	1–3	1–3
Petiole (length in mm)	5–15	5–10 (–22)	5–15	5–10
Leaf (shape)	Elliptic -obovate, lanceolate, subrhomboidal.	Elliptic, elliptic- oblanceolate or obovate.	Elliptic-lanceolate or obovate, somewhat rhomboidal.	Elliptic-obovate.
Leaf (apex)	Acute to shortly acuminate, emarginulate.	Protracted, blunt or acutish.	Shortly acute to attenuately acuminate, blunt and emarginulate.	Shortly attenuate, blunt or acutish.
Leaf (length in cm)	3.5–6	2–6 (–9)	3–6 (–11)	4–6.5
Leaf (width in cm)	2.5–4	1–2.5	2.5–4.5	2–3.5
Peduncle (length in cm)	1–2	1.4–2 (–4.5)	0.5–1	2–3
Spadix (length in cm)	8–12	5–7 (–10.5)	–12	5–7

This species belongs to Peperomiasubg.Micropiper (Miq.) Miq. (Frenzke et al. 2015).

##### Specimens examined.

**Peru. Dept. Cajamarca, prov. Santa Cruz, dist. Catache**: El Papayo, El Espinal, Monteseco, 600 m, [06°51'31.7"S, 79°06'46.8"W], 18 Jul 1984, *A. Sagástegui*, *E. García & S. Leyva 12354* (HUT, F 1960604, MO 3226489); Monteseco, 3 km NE, 1800 m, [06°50'42.3"S, 79°06'17.1"W], 5 May 1987, *J. Santisteban C. & J. Guevara B. 013* (HUT, F 1994832). Same place: 13 May 1987, *J. Santisteban C. & J. Guevara B. 055* (HUT 24750, F 1995336). Path in Monteseco Forest, 1350 m. [06°51'17.8"S, 79°06'33.3"W], 10 Oct 1993, *S. Leiva et al. 911* (HAO, F 2142594); Road from Cayaltí to La Florida, 549 m, 06°51'23.2"S, 79°09'31.1"W, 1 Mar 2009, *G.Mathieu & L. Symmank 161* (USM 255771); Road from El Espinal to La Florida, 519 m, 06°51'13.5"S, 79°09'45"W, 28 Aug 2022, *G. Pino et al. 3623* (USM 330876); Same road, 527 m, 06°51'16"S, 79°09'44"W, 28 Aug 2022, *G. Pino et al.* 3627 (USM 330877); Idem, 536 m, 06°51'18.6"S, 79°09'41.6"W, 28 Aug 2022, *G. Pino et al. 3632* (USM 330880); **Prov. San Miguel, dist. Niepos**: Road from Naranjo to La Florida, 1316 m, 06°53'08.6"S, 79°06'45.8"W, Jul 26 2020, *G. Pino & L.E. Alomía 3218* (USM 330868); Same road, 1291, 06°53'08.7"S, 79°06'49.1"W, Jul 26 2020, *G. Pino & L.E. Alomía 3219*; Same road, 1265 m, 06°53'04.6"S, 79°06'51.2"W, Jul 26 2020, *G. Pino & L.E. Alomía 3220*; Idem, 1213 m, 06°52'54.2"S, 79°06'51.9"W, Jul 26 2020, *G. Pino & L.E. Alomía 3221*; Idem, 1210 m, 06°52'53.6"S, 79°06'51.0"W, Jul 26 2020, *G. Pino & L.E. Alomía 3222*; Idem, 1176 m, 06°52'49.2"S, 79°06'52.5"W, Jul 26 2020, *G. Pino & L.E. Alomía 3223*; Idem, 1130 m, 06°52'44.1"S, 79°07'00.0"W, Jul 26 2020, *G. Pino & L.E. Alomía 3224*; Idem, 1118 m, 06°52'42.0"S, 79°07'01.1"W, Jul 26 2020, *G. Pino & L.E. Alomía 3225*; Idem, 1067 m, 06°52'32.9"S, 79°07'10.4"W, Jul 26 2020, *G. Pino & L.E. Alomía 3226*; Road from La Florida to Niepos, 1297 m, 06°53'6.9"S, 79°06'47.6"W, 28 Aug 2022, *G. Pino et al. 3642* (USM 330885).

#### ﻿5. Peperomia fraseri

Taxon classificationPlantaePiperalesPiperaceae

C.DC., J. Bot. 4: 134. 1866.

F8C29D4E-11A3-559E-941C-FCA0493A8F81

Fig. 5B

Peperomia rugosa Tafalla. Flora Huayaquilensis 1:12. 1989, nom. ined.
Peperomia resediflora Linden & André, Ill. Hort. 17: 135–137. fig. 26. 1870. [As resedaeflora] Type: (iconotype): Illustr. Hortic. 17: fig. 26.
Peperomia fraseri var. resedaeflora C.DC., Bull. Herb. Boissier 5: 703. 1897.Type: Based on Peperomiaresediflora Linden & André.
Peperomia fraseri var. peltata Yunck., Piperac. N. South Amer. 2: 740. 1950. Type: Ecuador, Loja, Horta-Naque, *Espinosa 859* (holotype: ILL, isotype: NY).
Peperomia treleasei Standl. & Steyerm., Fieldiana, Bot. 24(3): 273. 1952. Type: Guatemala (cultivated), *Steyermark 46298* (holotype: F).
Peperomia fraseri var. guayasana Trel., nom. herb. [Mathieu (2007)]. Type: Ecuador, Guayas, *Haught 3022* (holotype: US).

##### Type.

**Ecuador**, sine loco: Fraser s/n, (lectotype, designated by Mathieu and Callejas (2006: 351–352: G-DC!, isotypes: BM, G-DC)).

##### Distribution and habitat.

Ecuador (120–2100 m, prov. Loja, El Oro, Guayas, Chimborazo and Manabí). Peru, 500–1600 m in departments Tumbes, Piura, Lambayeque, and eastern Cajamarca along the Saña River valley, on rocky soil, shaded by trees and shrubs. This is the southernmost location of this species.

##### Specimens examined.

**Peru, dept. Lambayeque, prov. Chiclayo, dist. Oyotún**: road from Oyotún to El Espinal, 515 m, 06°48'30.3"S, 79°11'08"W, 26 Jul 2020, *G. Pino & L.E. Alomía 3236* (USM 325348); **Dept. Cajamarca, prov. Santa Cruz, dist. Catache**: road from Cayaltí to La Florida, 8.4 km from La Florida, 497 m, 06°50'51.6"S, 79°09'53.7"W, 1 Mar 2009, *G.Mathieu & L. Symmank 160* (USM 255770); road from Espinal to La Florida, 521 m, 06°51'14.2"S, 79°09'45"W, 28 Aug 2022, *G. Pino et al. 3636*; Same road, 527 m, 06°51'16"S, 79°09'44"W, 28 Aug 2022, *G. Pino et al. 3626*. Same road, 570 m, 06°51'14.1"S, 79°09'23.1"W, 26 Jul 2020, *G. Pino & L.E. Alomía 3231* (USM 325349).

##### Note.

This species belongs to Peperomiasubg.Panicularia Miq. (Frenzke et al. 2015).

#### ﻿6. Peperomia galioides

Taxon classificationPlantaePiperalesPiperaceae

Kunth, Nov. Gen & Sp. [Humboldt] 1: 58, tab. 17. 1815.

DF15871A-1A7F-5503-8028-AEABD814ACCD

Fig. 5C

Piper galioides Poir., Lam. Encycl. Meth. 4: Suppl. 4: 470. 1816. Type: Based on Peperomiagalioides Kunth.
Peperomia suaveolens Hamilton, Prodr. Pl. Ind. Occ.:2. 1825. Type: Cuba, (holotype: P- Herbarium Desvaux?).
Piper mollugo Willd. [Miquel] 1843. Type: Based on Peperomiagalioides Kunth.
Peperomia jamesoni Regel, Bull. Soc. Nat. Moscou 31: 544. 1858. Type: Ecuador, *Jameson 814* (holotype: G [not designated], isotypes: K, TCD).
Peperomia agapatensis C.DC., Prodromus 16: 455. 1869. Type: Perú, Junín, Agapata, *Lechler 1934* (holotype: G-DC; isotypes: G, GOET, K, MO, P, UPS, W).
Peperomia galioides Kunth var. longifolia C.DC., Prodromus 16 (1): 464. 1869. Type: Bolivia, *Mandon 1120* (syntypes: G-DC, B).
Peperomia galioides Kunth var. menkeana (Miq) C.DC., Prodromus 16 (1): 464. 1869. Type: Brazil, *Menke 125* (holotype: BR).
Peperomia galioides Kunth var. nigro -*punctulata* C.DC., Prodromus 16 (1): 464. 1869. Type: Brazil, Wawra 441, Ecuador, *Jameson s.n.* (syntype: K).
Peperomia galioides Kunth var. aprica Henschen. Nov. Act. Soc. Sci. Upsal. ser.3,8(2): 37. 1873. Type: Brazil, Minas Gerais, Caldas (Holotype: not designated).
Peperomia galioides Kunth var. umbrosa Henschen, Nov. Act. Soc. Sci. Upsal. ser.3,8(2):37. 1873. Type: Brazil, Minas Gerais, Caldas (Holotype: not designated).
Peperomia subcorymbosa Sodiro, Contrib. al Conoc. Fl. Ecuador. Monogr. 1 ed. 2:181. 1901. Type: Ecuador, Quito, El Alacatzo, *Sodiro s.n.* (holotype: Q, isotypes: G-DC, QPLS).
Peperomia galioides Kunth var. saxicola C.DC., Bull. Her. Boissier ser. 2,1: 360. 1901. Type: Brazil, *Schwacke 10279* (holotype: G).
Peperomia anisophylla C.DC., Bot. Jahrb. 40: 266. 1908. Type: Perú, Lima, Matucana, *Weberbauer 72* (holotype: B, isotype: G).
Peperomia galioides var. aromatica C.DC., Bot. Jahrb. Syst. 40: 266. 1908. Type: Peru. Ancash, Caraz. *Weberbauer 3227* (holotype: B [lost?]; isotype: G-DC).
Peperomia galioides Kunth var. minutifolia C.DC. Ann. Cons. Jard. Bot. Gen. 21: 262. 1920. Type: Ecuador, Quito, Nanegal, *Sodiro 2/52* (holotype: G-DC).
Peperomia granata Trel., Fedde Rep. Sp. Nov. 23: 24. 1926. Type: Cuba, Pico Turquino, *Ekman 14590* (lectotype: ILL [designated by Saralegui (2004)]; isolectotype: S).
Peperomia galiifolia Trel., Mem.N.Y. Bot.Gard. 7: 227. 1927. Type: Bolivia, La Paz, Pongo de Quime, *White 166* (holotype: NY).
Peperomia okarana Trel., Bull. Bot. Club. 55: 170. 1928. Type: Bolivia, La Paz, Larecaja, Okara, *Tate 970* (holotype: NY).
Peperomia artatiflora Trel., Flora of Peru [Macbride] 13(2): 23. 1936.Type: Perú, Palca, *Stevens 47* (holotype: ILL).
Peperomia brachyiula Trel., Flora of Peru [Macbride] 13(2): 26. 1936. Type: Perú, Lima, Matucana, *Macbride 129* (holotype: F, isotypes: G, ILL).
Peperomia ceapanana Trel., Flora of Peru [Macbride] 13(2): 28. 1936. Type: Perú, Cusco, Paucartambo, *Herrera 1468* (holotype:US).
Peperomia chillonensis Trel., Flora of Peru [Macbride]. 13(2): 30. 1936. Type: Perú, Lima, Canta, Obrajillo. *Pennell 14413* (holotype: F).
Peperomia dendroides Trel., Flora of Peru [Macbride]. 13(2): 38. 1936. Type: Perú, Junín, Chaglla, *Macbride 3640* (holotype: F, isotypes: G, ILL, US).
Peperomia distractiflora Trel., Flora of Peru [Macbride] 13(2): 40. 1936. Type: Perú, Junín, Challhuapuquio, *Stevens 214* (holotype: ILL).
Peperomia longispica Trel., Flora of Peru [Macbride] 13(2): 59. 1936. Type: Perú, Huánuco, Muña, *Macbride 3928* (isotype: BM).
Peperomia sanbuenaventurana Trel., Flora of Peru [Macbride] 13(2): 91. 1936. Type: Perú, Lima, Canta, San Buenaventura. *Pennell 14566* (holotype: PH).
Peperomia trullifolia Trel., Flora of Peru [Macbride] 13(2): 102. 1936. Type: Perú, Cusco, Ollantaytambo, *Cook 401* (holotype: US).
Peperomia inaequalifolia Ruiz & Pav. var. galioides (Kunth) Pino, Avonia 28(2): pages 45–49 (f.16–25). 2010. Type: Based on Peperomiagalioides Kunth.

##### Type.

**Colombia**, dept. Cundinamarca, prov. Tequendama: “Crescit in montanis Regni Novogranatensis, juxta cataractam Tequendamae, alt. 1300 hexap. Floret Augusto” [Grows in mountains of Colombia, close to the Tequendama Waterfall, 2400 m, Flowers in August], *Humboldt s/n*, (isolectotype, designated by Saralegui (2004: 341: B-W [00762])).

##### Distribution and habitat.

Montane forests of Mexico, Central America, Venezuela, Colombia, Ecuador, Peru, Bolivia, and Brazil, on rocks or terrestrial, always above 2200 m.

##### Note.

This species belongs to Peperomiasubg.Micropiper (Miq.) Miq. (Frenzke et al. 2015).

##### Specimens examined.

**Peru, dept. Cajamarca, prov. Santa Cruz, dist. Catache**: Udima, herbácea, creciendo sobre piedra, [herb growing on rocks] 2500 m, [06°49'38.3"S, 79°06'01.1"W], 20 May 1987, *J.Santisteban C. & J. Guevara B. 087* (HUT, F1995293). **Prov. San Miguel, dist. Niepos**: Road from La Florida to Niepos, 2240 m, 06°54'49"S, 79°08'17.7"W, 28 Aug 2022, *G. Pino & L.E. Alomía 3647* (USM 330888); Dist. Calquis: Taulis Playa (Agua Blanca) 2650 m, [06°56'06"S, 78°59'23"W], 3 Jul 1986, *J. Mostacero et al. 1143* (HUT 22520, MO).

#### ﻿7. Peperomia haematolepis

Taxon classificationPlantaePiperalesPiperaceae

Trel., Field. Mus. Pub. Bot. [Macbride] 13(2):52. 1936.

7FEC2363-F413-51E0-804B-15002FF818EE

Fig. 5D

##### Type.

**Peru**, dept. Junín, prov. Chanchamayo, dist. San Ramón: Hacienda Chalhuapuquio, *Stevens 212* (holotype: ILL).

##### Distribution and habitat.

Plants are reported from Brazil, the Guyanas, Venezuela, Colombia, Ecuador, and Peru from 1000 to 2000 m, epiphytic in montane forests shaded by the canopy. Most of the collections in Peru are from the Amazon Basin; this is the first report for a Pacific Ocean draining Andean valley.

##### Notes.

This species belongs to Peperomiasubg.Oxyrhynchum (Dahlst.) Samain (Frenzke et al. 2015).

##### Specimen examined.

**Peru, dept. Cajamarca, prov. Santa Cruz, dist. Catache**: Upper Río Zaña Valley, ca. 5 km above Monteseco, on the path below the campsite, lower reaches of evergreen tropical montane forest, 1450 m, [06°51'10.8"S, 79°06'09.4"W], 19 Mar 1986, *M.O. Dillon & A. Sagástegui 4425* (US 3338748, F, GB 0167869, HUA 49003).

#### ﻿8. Peperomia hispidula

Taxon classificationPlantaePiperalesPiperaceae

(Sw.) A. Dietr., Sp. Pl. 1: 165. 1831.

4B0B2059-FF56-5528-A28C-90FA087A407E

Fig. 5E

Piper hispidulum Sw., Prodr. 15. 1788. Type: Jamaica, Swartz s.n. (holotype not designated: S) [Basionym for Peperomiahispidula (Sw.) A.Dietr.] .
Acrocarpidium hispidulum Miq., Syst. Piperac. 54. 1843 Type: Based on Piperhispidulum Sw.
Acrocarpidium sellowianum Miq., Syst. Piperac. 55. 1843 [as ‘sellovianum’] Type: Brazil, Brasilia, Sellow s.n. (holotype: B [lost?], lectotype: U [designated Zanotti et al. (2012: 133)], isolectotypes: G, K, W).
Peperomia tenera Miq., Fl. Bras. [Martius] 4(1): 19. 1852. Type: Based on Acrocarpidiumsellowianum Miq.
Peperomia muscophila C.DC., Journ. Bot. 4: 133. 1866. [as ‘muscophylla’] Type: Mexico, Tafalla s.n (holotype: G; isotypes: G-DC).
Peperomia hispidula C.DC., Prodr.16:1:397. 1869. Based on Piperhispidulum Sw.
Peperomia hispidula var. sellowiana (Miq.) Dahlst. Kongl. Svenska Vetenskapsakad. Handl. 33(2): 14 . 1900. Type: Based on Acrocarpidiumsellowianum Miq.
Peperomia hispidula var. swartziana Dahlst., Kongl. Svenska Vetenskapsakad. Handl. 33(2): 13.1900. Type: Based on Peperomiahispidula C.DC.
Peperomia hispidula var. swartziana f. barbensis Dahlst., Kongl. Svenska Vetenskapsakad. Handl. 33(2): 14 .1900. Type: Costa Rica, Hoffmann 54 (holotype: B [lost?]).
Peperomia perhispidula C.DC., Bot. Jahrb. Syst. 40: 257. 1908). Type: Peru, Huacapistana, Weberbauer 2014 (holotype: B, F G-DC).
Peperomia hispidula var. muscophila (C.DC.) C.DC., Candollea 1: 335 .1923. Type: based on Peperomiamuscophila C.DC.
Peperomia hispidula var. perhispidula Trelease & Yuncker, Candollea 1: 335. 1923. Type: Based on Peperomiaperhispidula C.DC.
Peperomia hispidula var. ellipticifolia Trelease & Yuncker, Piper. North. S. Amer. 2: 705. Pl. 626. 1950. Type: Colombia, Suratá, Killip & Smith16580 (holotype: GH; isotypes: ILL, MA, NY, US).

##### Type.

**Jamaica**, In nemorosis humidis montium altissimorum coeruleorum Jamaicae. [In shaded moist elevated forest on Blue Mountains Peak of Jamaica, 2200 m, 18°6'N, 76°40'W], *Swartz s.n.* (holotype not designated: S!)

##### Distribution and habitat.

Terrestrial on humus near water courses, shaded, 1500–3000 m, in evergreen montane forests of West Indies, Mexico, Central America, and South America (except Chile). All the other collections in Peru are from the eastern slopes of the Andes; this is the only report for the western slopes.

##### Note.

This species belongs to Peperomiasubg.Hispidula Frenzke & Scheiris (Frenzke et al. 2015).

##### Specimen examined.

**Peru, dept. Cajamarca, prov. Santa Cruz, dist. Catache**: Al norte del Chorro Blanco (Monteseco), borde de acequia [North of Chorro Blanco (Monteseco), border of stream] 1500 m, [06°50'58.6"S, 79°05'53.7"W], 20 Jan 1989, *S. Leyva G. 008* (F 2016256).

#### ﻿9. Peperomia inaequalifolia

Taxon classificationPlantaePiperalesPiperaceae

Ruiz & Pav., Flora Peruviana & Chilensis 1: 30, plat. 46 fig.a. 1798.

93D4BB96-99BC-5502-AFA2-F9584E8D5D5C

Fig. 5F

Piper inaequalifolium Vahl., Enum. Pl. 1: 355. 1804. Type: Based on Peperomiainaequalifolia Ruiz & Pav.
Piper aromaticum Willd., Enum. Pl. Hort. Berol. Suppl.: 3. 1813. Type: Not mentioned.
Peperomia chrysotricha Miq., Syst. Pip.:163. 1843. Type: Dombey 933 (holotype: P; isotype: U).
Peperomia parvula Sodiro, Piperac. Ecuator. 1: 139. 1900. Type: Sodiro s/n (Holotype not designated: G, P, Q).
Peperomia fasciculata Sodiro, Contrib. al Conoc. Fl. Ecuador Monog.1 ed. 3: 181. 1901. Type: Based on Peperomiaparvula Sodiro.
Peperomia galioides Kunth var. minutifolia C.DC., Ann. Cons. Jard. Bot. Genève 21: 262. 1920. Type: Ecuador, Valle Nonegal. A. Sodiro 2/52 (holotype: G-DC).
Peperomia atocongona Trel., Publ. Field Mus. Nat. Hist., Bot. Ser. [Macbride J.F.] 13(2): 24. 1936. Type: Peru. Lima: Atocongo. F.W. Pennelll 14752 (holotype: PH).
Peperomia limaensis Trel., Publ. Field Mus. Nat. Hist., Bot. Ser. [Macbride J.F.] 13(2): 58. 1936. Type: Peru. Lima: San Gerónimo. J.F.Macbride 5920 (holotype: F).
Peperomia pseudogalapagensis Trel., Publ. Field Mus. Nat. Hist., Bot. Ser. [Macbride J.F.] 13(2): 79. 1936. Type: Peru. Lima: San Gerónimo. Wilkes Expl. Exped. 5920 (holotype: GH; isotype: K).

##### Type.

**Peru**, dept. Lima, prov. Lima, dist. Rimac: “Habitat Limae in collibus altis in Amancaes copiosa, dicitur Congona Zimarrona” [Grows in Lima in high hills, abundant in Loma de Amancaes, it is called Congona Cimarrona], Ruiz & Pavón s/n: MA [29573]

##### Distribution and habitat.

Coastal valleys of Peru and Galápagos (Ecuador) between 200 and 2000 m, in dry forests, almost always epiphytic on trunks, occasionally on rocks.

##### Note.

This species belongs to Peperomiasubg.Micropiper (Miq.) Miq. (Frenzke et al. 2015).

##### Specimens examined.

**Peru, dept. Cajamarca, prov. Santa Cruz, dist. Catache**: Monteseco, 3 km NE, epífito, sobre tronco de árbol [epiphytic, on tree trunk] 1850 m, [06°50'41.4"S, 79°06'14.4"W], 5 May 1987, *J. Santisteban C. & J. Guevara B. 004* (HUT 24701, F1995288); Arriba de Monteseco, sobre árbol de *Erythrina* [Above Monteseco, on *Erythrina* tree] 1350 m, [06°51'17.8"S, 79°06'33.3"W], 10 Oct 1993, *S. Leyva et al. 904* (HAO, F 2165067) **Prov. San Miguel, dist. Niepos**: Road from La Florida to Monte Seco (Naranjo), 15.6 km from La Florida, 1700 m, 06°54'08"S, 79°08'15"W, 1 Mar 2009, *G. Mathieu & L. Symmank 166*. (USM 255775). Road from Naranjo to La Florida, 1316 m, 06°53'08.6"S, 79°06'45.8"W, 26 Jul 2020, *G. Pino & L.E. Alomía 3227* (observed). Same road, 1291 m, 06°53'08.7"S, 79°06'49.1"W, 26 Jul 2020, *G. Pino & L.E. Alomía 3228* (observed). Idem. 1265 m, 06°53'04.6"S, 79°06'51.2"W, 26 Jul 2020, *G. Pino & L.E. Alomía 3229* (observed). Road from La Florida to Niepos, 1297 m, 06°53'06.9"S, 79°06'47.6"W, 28 Aug 2022, *G. Pino & L.E. Alomía 3640*. (USM 330884).

#### ﻿10. Peperomia microphylla

Taxon classificationPlantaePiperalesPiperaceae

Kunth, Nov. Gen & Sp. [Humboldt] 1: 57, tab. 15, fig. 2. 1815.

10521FB5-BAB2-5EBF-93DB-BFF0E64B2E5D

Fig. 5G

Piper microphyllum (Kunth) Poir., Lam. Encycl. Meth. Suppl. 4: 469. 1816. Type: Based on Peperomiamicrophylla Kunth.
Peperomia aphylla C.DC., Prodr. 16(1): 456. 1869. Type: Colombia. Quindío, *Jameson 690* (holotype: G; isotypes: BM, K).
Peperomia gilbertii Trel., Publ. Field Mus. Nat. Hist., Bot. Ser. [Macbride J.F.] 13(2): 50.1936. Type: Peru. Cusco: Ollantaytambo, *Cook & Gilbert 740* (holotype: US).
Peperomia chachopoana Trel., Cat. Fl. Venez. 1:244. 1945, Nom. Nud. Type: Venezuela. Mérida. (2800 m) *Pittier 13165* (holotype: NY; isotypes: F, M, NY, US).

##### Type.

**Colombia**, dept. Quindío: “Crescit in lapidosis frigidis Andium Quinduensis, juxta El Boquerón del Páramo, alt. 1650 hexap. Floret Octobri” [Grows in cold rocky places of the Andes of Quindiu, close to El Boquerón del Páramo, 3000 m, Flowers in October], Humboldt s/n, (lectotype, designated by Mathieu and Callejas (2006: 341: B!; isolectotype: P)).

##### Distribution and habitat.

Montane forests of the Andes of Venezuela, Colombia, Ecuador, and Peru, on rocks or more frequently epiphyte.

##### Note.

This species belongs to Peperomiasubg.Micropiper (Miq.) Miq. (Frenzke et al. 2015).

##### Specimen examined.

**Peru, dept. Cajamarca, prov. Santa Cruz, dist. Catache**: ca. 4.2 Km (por aire) [in a straight line] NE Monteseco, 2500 m, [06°49'47.1"S, 79°06'22.4"W], 20 May 1987, *J. Santisteban C. & J. Guevara B 084* (HUT 24800, F1995295).

#### ﻿11. Peperomia pilocarpa

Taxon classificationPlantaePiperalesPiperaceae

Pino, Samain & L.E. Alomía sp. nov.

40575DCB-4F94-5CF2-8E07-2218A04617A2

urn:lsid:ipni.org:names:77317652-1

Fig. 7A–G

##### Type.

**Peru**, Dept. Cajamarca, prov. San Miguel, dist. Niepos: road from La Florida to Niepos, 1613 m, 06°54'03.3"S, 79°07'33.5"W, 26 Jul 2020, *G. Pino & L.E. Alomía 3217* (USM 333265!); Same collection, 28 Aug 2022, *G. Pino et al. 3646* (USM 333266!).

**Figure 7.**
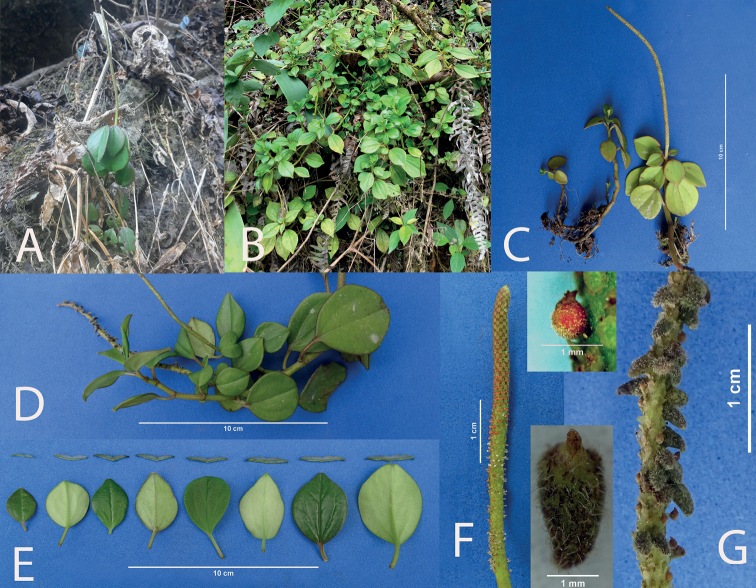
*Peperomiapilocarpa***A** plant in habitat with assurgent stems **B** decumbent plant **C** young plants showing terminal inflorescence **D** plant *ex-situ* in anthesis showing normal inflorescence and inflorescence with galls **E** detail of leaves **F** spadix at the beginning of anthesis **G** mature spadix with galls and seeds. Details: (above, normal seed; below, gall with trichomes).

##### Diagnosis.

Perennial, semi-succulent, prostate caespitose herb similar in habit to *P.cacaophila*, but differs mainly in leaf shape, which is widely obovate to elliptic or rotundate and sometimes slightly acuminate, compared to the ovate lamina and constantly acuminate apex of this species, leaves are flatter and less succulent, slightly puberulous compared to the canaliculate, glossy leaves of *P.cacaophila*. Stems are not terete as in this species but with two low prominent wings. Seeds are dimorphic, normal seeds are very similar in shape to the seeds of *P.sagasteguii*, modified seeds (probably galls) are conspicuous, large up to 2.5 mm long, bright green to brownish, densely covered with white trichomes.

##### Description.

Perennial semi succulent terrestrial or semiepiphytic ***herb***, living in the shade and on abundant decayed vegetal matter, 10–15 cm tall, up to 25 cm when flowering. ***Roots*** basal and from basal nodes, very fibrous, light gray, 0.2–0.3 mm diam., 1–3 cm long. ***Stem*** mainly prostate, straight, procumbent when vegetative and decumbent sometimes at tips, up to 45 cm long or more, subterete, 2.5–3.5 mm diam. at the base, gradually tapering to 1.5–2 mm at the apex, dull olive green, reddish where exposed, with two not very prominent longitudinal wings decurrent with leaf petioles, internodes 1–5 cm, rarely with alternate branches from the base or every 10–15 cm. ***Leaves*** alternate, spirally attached one per internode, glabrous to slightly puberulous, present all along the stem; petiole circular in section, 0.8–1.5 cm long, 1–1.5 mm diam., with two lateral not prominent wings decurrent with stem, straight or slightly recurvate, lamina flat, widely obovate to oblong, (2–) 3–6 cm long, 1–3.5 mm thick, (1–)1.5–2.5 cm wide at distal third, (1.5–)2.8–3.8 cm wide at the middle, (1–)2–3 cm wide at proximal third, apex acute, obtuse in some leaves and slightly acuminate in larger leaves, subemarginate at tip, base rounded; adaxially dull green, 3-palmatinerved, nerves slightly depressed; margin completely entire; abaxially light green, obscurely 3-palmatinerved, central nerve and proximal thirds of lateral nerves somewhat darker in color. ***Inflorescence*** a single terminal spadix appearing from November to December; peduncle terete or slightly funnel-shaped distally, sometimes reddish and obscurely furrowed, 7–12 mm long, 2–2.5 mm diam., with a small oval bract at the base, similar to leaves, ***rachis*** 6–10 cm long, 1.5–2.5 mm diam., light green. ***Floral bracts*** narrowly oval to subpentagonal, subacute, light green, 0.6–0.7 mm long, 0.5–0.6 mm wide. ***Stamens***: filaments transparent, 0.2 mm diam., 0.4–0.5 mm long, anthers ovoid, 0.4–0.45 mm long, 0.25–0.3 mm wide, bright red at first, then white. Normal ***fruit*** globose, 0.7–0.8 mm long, 0.6–0.7 mm diam., brown, covered of white 0.05 mm long whitish papillae up to distal third of fruit, style conical, light brown. Modified ***fruit***, 2–2.5 mm long, 1.2–1.5 mm diam., bright green to brownish, minutely and densely covered with white 0.1–0.2 long trichomes, style prominent, bright green, 0.2–0.25 mm long, 0.3–0.4 mm diam., stigma dark, ripening from February to July.

##### Distribution and habitat.

Plants grow from 1500 to 1600 m of the middle course of the Saña River valley, in the remnants of montane forest, mostly epiphytic.

##### Phenology.

Inflorescences appear from October to March; fruits ripen from November to April.

##### Etymology.

The epithet recalls the hairy surface of the modified fruits of this species, from the Latin *pilus* (hair) and Greek *καρπός* (fruit).

##### Notes.

This specimen at first was considered a probable hybrid because it shows intermediate features between *P.emarginulata* and *P.cacaophila*. However, although those two species share their habitat to some extent they are found at lower altitudes and never close to *P.pilocarpa*. Instead, this species appears within the range of *P.vivipara*, but it seems not to be related to it. Another interesting fact is that most fruits are modified to form hairy galls. The etiology of these galls remains unknown.

This species belongs to Peperomiasubg.Micropiper (Miq.) Miq. (Frenzke et al. 2015).

##### Additional specimen examined.

**Peru, dept. Cajamarca, Prov. San Miguel, dist. La Florida**: Road from Monteseco to Chorro Blanco, 1545 m, 6°50'51.3"S, 79°06'23.5"W, 10 Feb 2023, *G. Pino & L.E.Alomía 3830*, (USM 333267).

#### ﻿12. Peperomia riosaniensis

Taxon classificationPlantaePiperalesPiperaceae

Hutchison ex Pino, Samain & L.E. Alomía sp. nov.

A0824C09-D189-51CF-BC26-CB35C06A637C

urn:lsid:ipni.org:names:77317653-1

Fig. 8A–H

##### Type material.

**Peru**, [**dept. Lambayeque, prov. Chiclayo, dist. Oyotún**]: road to Hacienda Taulis, ca. 80 km up the Río Saña from the Pan American Highway, 500 m, [06°51'23"S, 79°09'31"W], 28 Aug 1964, *P. C. Hutchison 6308* (holotype: UC[1298471]!, isotypes US [2485139]!, USM [Not found]).

**Figure 8.**
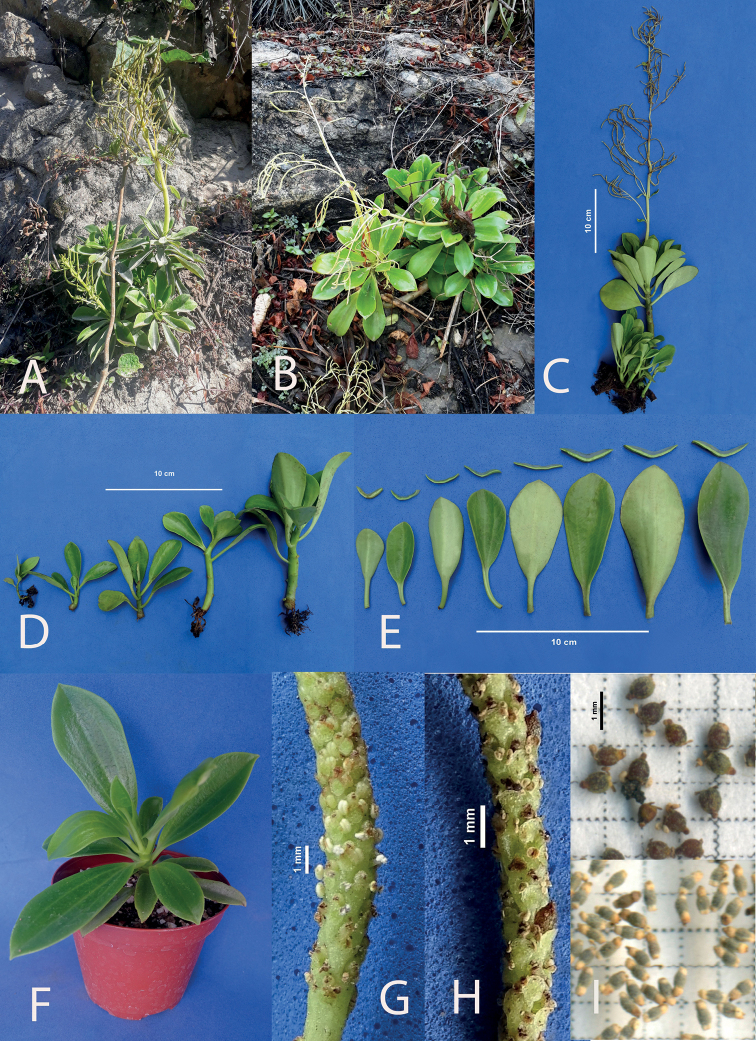
*Peperomiariosaniensis* Pino, Samain & L.E. Alomía, sp. nov. **A, B** plant in habitat **C** plant *ex situ***D** early stages of development **E** detail of the leaves **F** young plant in cultivation **G** spadix in anthesis **H** mature spadix with fruits **I** comparison of the fruits of *P.riosaniensis* (above) and *P.palmiformis* (below).

##### Diagnosis.

Perennial, succulent terrestrial herb similar to *P.palmiformis* Pino & Samain, differs in inflorescences that have wider panicles, with more (30–60 compared to 16–40) and shorter spadices (1–5 cm long compared to 2–8 cm long), leaves attached all along the stem, slightly longer (6–9 cm compared to 5–7 cm), more succulent, canaliculate instead of flat, with longer petioles (2 cm compared to 0.7 cm), lamina wider at proximal third (1.2–1.8 cm compared to 0.5–1.2), margin entire distally instead of serrate, fruits larger (0.9 × 0.6 mm compared to 0.4 × 0.3 mm), widely ovoid instead of narrowly ovoid.

##### Description.

Perennial, succulent, terrestrial ***herb***, living in gorges or the shade of other plants, (4–) 9–18 cm tall, up to 50 cm tall when flowering. Roots fibrous, light gray, 0.7–0.4 mm diam., 2–4 cm long. Stem mainly erect or shortly decumbent at base, straight, terete, slightly puberulous, 0.7–1.2 cm diam. at the base, gradually tapering to 4–8 mm at the apex, bright green, brownish at the base, with reniform leaf scars spirally displayed every 3–8 mm, 1.5–2 mm × 3–4 mm, brownish; very rarely branching dichotomously from the distal 10 cm, but mostly with 4–6 branches from the base, resulting in a caespitose plant. ***Leaves*** alternate, glabrous, spirally inserted, present all along the stem or in the distal third of the main stem or branches under dry conditions, young leaves smaller, narrower, very succulent, induplicate or canaliculate, older leaves less succulent; petiole reniform in section, gradually continuing with leaf, 0.5–2 cm long, 2.5–3 mm wide, 1.5–2 mm thick, lamina convex to induplicate, narrowly obovate, (3–) 6–9 cm long, 1.2–2.4 mm thick, (1.4–)2–3 cm wide at distal third, (1.4–)1.8–3 cm wide at the middle, (1–)1.4–1.8 cm wide at proximal third, apex subacute, distal third slightly recurved, base cuneate; adaxially bright glossy green, convex to slightly induplicate, obscurely 3–5-palmatinerved, nerves lighter colored; canaliculate or markedly induplicate, only the central nerve is visible in young leaves; margin entire; abaxially pale green, obscurely 3–5-palmatinerved, central nerve subcarinate, the others rather depressed. ***Inflorescence*** is an open terminal panicle with 30–60 alternate spadices emerging solitary or from secondary axes with 2–7 spadices, each born from 10–20 nodes appearing 5–10 cm from the base of the central axis; each node with a basal lanceolate acute bract 0.4–2 cm long, 0.3–0.7 cm wide, bright green; central axis 30–40 cm long, 3–6 mm diam. at the base, gradually tapering to 1 mm, terete, longitudinally furrowed, very light green; ***peduncle*** terete or slightly funnel-shaped distally, 3–10 mm long, 0.7–1.2 mm diam., each with a 2 mm long, 0.5 mm wide oval bract at the base, deciduous; ***rachis*** 1–5 cm long, 1.2–1.6 mm diam., light green. ***Floral bracts*** broadly oval to round, subacute, light green, 0.5–0.6 mm diam. ***Stamens***, filaments 0.4 mm long, 0.2 mm diam., transparent, anthers white ovoid, 0.4–0.5 × 0.3 mm. ***Fruit*** an ovoid berry, 0.85–0.9 mm long, 0.55–0.6 mm diam., bright brown to olive green, minutely papillate, pedicel inconspicuous, dry anthers attached, style prominent, ovoid, bright brown, 0.15–0.2 mm long, 0.15–0.2 mm diam., stigma lighter colored.

##### Distribution and habitat.

Plants grow from 450 to 600 m, the lowest and driest layer of the area studied, along the Saña valley between the towns of Oyotún and La Florida in the departments Lambayeque and Cajamarca, on rocky soil, in thickets shaded by shrubs and supplied with moisture from nearby water courses.

##### Phenology.

Inflorescences appear from June to August; fruits ripen from July to September.

##### Etymology.

The epithet was coined by Hutchison in 1967 after the Saña river, “Río Saña” in Spanish, which is Latinized to “riosaniensis” in the same way *Rauhocereusriosaniensis* was described by Backeberg. As Hutchison only labeled the herbarium sheets and never published this name it has been considered a *nomen herbariorum* (Mathieu 2007).

##### Notes.

The closest species of *Peperomiariosaniensis* is *P.palmiformis* Pino & Samain (Pino et al. 2012) The web page “Peperomia.net” (http://www.peperomia.net/repertorysearch.asp) indicates that this taxon is merely a synonym of the latter. We compared both species in detail and the main differences between *P.riosaniensis* and *P.palmiformis* are resumed in Table 3.

**Table 3.** Comparison of the main differences between *P.riosaniensis* and *P.palmiformis*.

Features	*P.riosaniensis*	*P.palmiformis*
Height (vegetative in cm)	9–18	15–20
Distribution of leaves	All along the stem	Mainly on distal third.
Branches	Very common, basal, rarely dichotomous distally.	Rarely branched from the base, distal dichotomous branches.
Leaf (petiole)	0.5–2 cm long	3–7 mm long
	2.5–3 mm wide	1.2–1.8 mm wide
	1.5–2 mm thick	1–1.5 mm thick
Leaf (shape)	Canaliculate to induplicate, narrowly obovate. Distal third slightly recurved.	Mainly flat, occasionally slightly convex on both sides, very narrowly obovate.
Leaf (length in cm)	6–9	5–7
Leaf (thickness in mm)	1.2–2.4	0.5–3
Leaf (width)	2–3 cm wide at distal third	2–2.5 cm wide at distal third
	1.8–3 cm wide at the middle	1–2 cm wide at the middle
	1.4–1.8 cm wide at proximal third	0.5–1.2 cm wide at proximal third
Leaf (margin)	Completely entire.	Constantly serrate at distal third.
Inflorescence	Open panicle	Narrow panicle
	10–20 branches,	10–16 upright branches,
	30–60 spadices.	16–40 spadices.
Central axis	30–40 cm long	25–30 cm long
	3–6 mm diam. at base	3.5–5 mm diam. at base
Rachis of the spadix	1–5 cm long, 1.2–1.6 mm diam.	2–8 cm long, 1–1.4 mm diam.
Fruit (shape/color)	Ovoid, bright brown to olive green.	Narrowly ovoid, light olive green
Fruit (length in mm)	0.85–0.9	0.35–0.45
Fruit (diam. in mm)	0.55–0.6	0.25–0.35
Fruit (style)	Wide ovoid, bright brown,	Long ovoid to balaniform, bright orange,
	0.15–0.2 mm long,	0.25–0.30 mm long,
	0.15–0.2 mm diam.	0.22–0.28 mm diam.

While *P.palmiformis* grows from 800 to 900 m along the Utcubamba valley from Bagua to Pedro Ruiz in department Amazonas, Peru, *P.riosaniensis* grows 300 m lower. Nevertheless, the temperatures in the habitats of both species average 25–30 °C during the day and 18–22 °C at night, *P.riosaniensis* grows at 200 km distance from *P.palmiformis*, on the dry, xerophytic western slopes of the Andes, with 25 mm rain per month average in summer and 0–4 mm in winter (Clima.com: https://www.clima.com/peru/cajamarca/florida). *Peperomiapalmiformis* lives on the flatter eastern slopes of the Andes, along the lower basin of the Utcubamba river, in a dry valley but already under the influence of the Amazon Forest, with nearly 60 mm rain per month in summer and 5 mm monthly in winter (Clima.com). Both species look similar from far away, but *P.riosaniensis* is slightly shorter when vegetative; its inflorescences have relatively wider panicles, with more horizontal branches and more spadices that are relatively shorter. Although leaves look very much alike when pressed, the new species has longer and thicker petioles, longer leaves that are not as narrow and succulent, wider at the proximal third, making its margin convex, compared to the concave margin of *P.palmiformis* at this segment. The new species also has induplicate or canaliculate leaves, adaxially slightly darker and glossier, frequently carinate beneath and slightly recurved at the distal third, compared to the straight, flat, mostly biconvex succulent leaves of *P.palmiformis*, mainly due to its more developed fenestra. The serrate distal margin of the leaves observed in *P.palmiformis* similar to *P.erosa* Hutchison ex Pino (Pino et al. 2012) has never been observed in the new species. Fruits are entirely different, more globular, larger, thicker, and papillate in *P.riosaniensis*, frequently with dry anthers attached, and lacking the conspicuous bright orange persistent basal pedicel. *Peperomiapalmiformis* fruits also have a larger, bright orange style, with a bulge that is sometimes even thicker than the fruit itself. These morphological differences are supported by the fact that in a recent phylogenetic study, *P.riosaniensis* is located in a different branch from *P.palmiformis* that is more related to *P.columella*, *P.ferreyrae*, *P.mathieui*, and *P.columnaris* from the Utcubamba valley (Frenzke et al. 2015). All these species mentioned belong to PeperomiasubgenusFenestratae Pino (Frenzke et al. 2015).

##### Additional specimens were examined.

**Peru, dept. Lambayeque, prov. Chiclayo, dist. Oyotún**: road from Oyotún to El Espinal, on the trail to waterfall Velo de la Novia, 515 m, 06°48'30.3"S, 79°11'08"W, 26 Jul 2020, *G. Pino & L.E. Alomía 3237* (USM 330870); same road, 520 m, 06°48'27.4"S, 79°11'04.7"W, 26 Jul 2020, *G. Pino & L.E. Alomía 3238* (USM 330871); **Dept. Cajamarca, prov. Santa Cruz, dist. Catache**: Road from El Espinal to La Florida, 582 m, 06°51'13.7"S, 79°09'16.5"W, 26 Jul 2020, *G. Pino & L.E. Alomía 3230* (USM 330869); Road from Cayaltí to La Florida, 8.4 km from La Florida, 492 m, 06°50'51,6"S, 79°09'53,7"W, 1 Mar 2009, *G. Mathieu & L. Symmank 2009-167* (BR, GENT, USM 258833!) Same road, 591 m, 06°51'13.4"S, 79°09'18.4"W, 28 Aug 2022, *G. Pino et al. 3637* (USM 330882) **prov. San Miguel, dist. La Florida**, Puente el Papayo, on slopes at the borders of the roadside, 18 Jul 1982, *S. Llatas Quiroz 861* (F 1931956, NY).

#### ﻿13. Peperomia rotundata

Taxon classificationPlantaePiperalesPiperaceae

Kunth. Nov. Gen & Sp. [Humboldt] 1: 67, tab.12. 1815.

4C10D00B-4225-5684-BE41-9260C18E76F4

Fig. 5H

Piper rotundatum (Kunth) Poir., Lam. Encycl. Meth. Suppl. 4: 468. 1816. Type: Based on Peperomiarotundata Kunth.
Piper villosum Rich. mss. in herb. Francov. in DC. Prodr. 16(1):442. 1869. Type: Based on Peperomiarotundata Kunth.

##### Type.

**Colombia**, dept. Cauca: “Crescit in excelsis regni Novogranatensis, inter Pansitara et vallem Yacanocatu, alt. 980 hexap. Floret Novembri.” [Grows in elevations of the Kingdom of New Granade, between Pacitará and the Yacanocatu valley, at 1800 m, Flowers in November], Humboldt s/n, (holotype: B [lost?]; isolectotype: P!)

##### Distribution and habitat.

Montane forests of Venezuela, Colombia, Ecuador, Peru, and Bolivia: rare, but uniformly distributed, terrestrial in shaded places growing on humus.

##### Note.

This species belongs to Peperomiasubg.Micropiper (Miq.) Miq. (Frenzke et al. 2015).

##### Specimen examined.

**Peru, dept. Cajamarca, prov. San Miguel, dist. Unión Agua Blanca**: El Tingo, camino a Taulis, [on the way to Taulis] 3175 m, [06°59'26"S, 79°01'21"W], 18 Feb 2000, *E. Rodríguez et al. 2366* (HUT 37499, F, M, MO).

#### ﻿14. Peperomia sagasteguii

Taxon classificationPlantaePiperalesPiperaceae

Pino, Samain & L.E. Alomía sp. nov.

62EC15CC-F717-5054-8C6A-E3F726595B43

urn:lsid:ipni.org:names:77317654-1

Fig. 9A–G

##### Type.

**Peru**, dept. Cajamarca, prov. Santa Cruz, dist. Catache: Monteseco, 1 Km above, on the path to Chorro Blanco, 1353 m, [06°51'17.8"S, 79°06'33.2"W], 16 Mar 1986, *M.O. Dillon & A. Sagástegui 4315a* (holotype: F [1978653]!).

**Figure 9.**
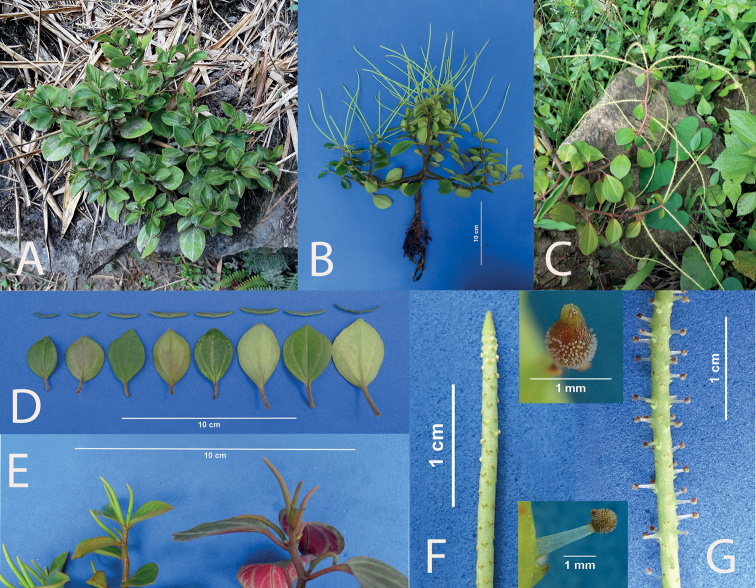
*Peperomiasagasteguii* Pino, Samain & L.E. Alomía, sp. nov. **A** plant in habitat in the dry period **B** plant *ex situ* in anthesis **C** plant in habitat with inflorescences **D** detail of the leaves **E** comparison of *P.sagasteguii and P.trinervis* at the beginning of anthesis **F** detail of immature spadix **G** mature spadix showing long pedicels and fruits. (Details: Above, mature fruit; Below, fruit standing on long pedicel).

##### Diagnosis.

Perennial, semi-succulent, terrestrial, epiphytic, or saxicolous herb similar to *P.trinervis*, differs in stems more pubescent, dark red to olive green instead of bright red, leaves with a longer and wider petiole (7–17 long × 1.8–2 wide compared to 3–10 long × 1.2–1.5 wide), lamina more succulent, green adaxially and light green-pinkish abaxially instead of olive green with gray nerves adaxially and red-purple abaxially; spadices longer (4–20 cm compared to 10 cm long), but narrower.

##### Description.

Perennial, semi-succulent, terrestrial, epiphytic, or saxicolous ***herb***, living in the shade of other plants, 10–30 cm tall, up to 45 cm tall when flowering. Roots fibrous, around 1 mm diam., transparent-reddish, 2–6 cm long, emerging from basal nodes and stolons. ***Stem*** stoloniferous at the base, then assurgent, thicker below, straight erect, terete, dark red to olive green, 0.4–0.6 cm diam. at the base, gradually tapering to 1.4–1.6 mm at the apex, readily branched alternately, internodes 2.5–3 cm at the base gradually descending to 0.8–1 cm towards the apex, surface crisp-pubescent, trichomes whitish, around 0.5 mm long at base, slightly shorter upwards. ***Branches*** 5–9, irregularly alternate, sometimes pseudo-opposite below, emerging almost horizontally mainly from the distal half, 7–15 cm long in the vegetative state. ***Leaves*** alternate, elliptic, elliptic obovate to subrotundate; petiole terete, same surface and color as stem, 0.7–1.7 cm long, 1.8–2 mm wide, slightly channeled above; lamina slightly convex to flat, 2.5–4 cm long, 1–2 mm thick, 1.6–2.8 cm wide, apex acute to attenuately subacuminate, base cuneate to sub truncate; adaxially bright glossy green, convex, 3-palmatinerved, nerves depressed; margin entire; abaxially pale green to pinkish, 3-palmatinerved, nerves elevated. Young leaves can be pinkish adaxially. Both sides are puberulous. ***Inflorescence*** terminal in an umbel of 1–3 spadices and sometimes 1–2 individual spadices from upper leaf nodes, umbel with 1–3 basal bracts at the base and lateral spadices with opposite bracts at base similar to leaves but much smaller; ***peduncle*** terete, light green, 4–6 mm long, 1.2–1.8 mm diam.; ***rachis*** 4–15 (–20) cm long, 1.5–1.7 mm diam., light green. ***Floral bracts*** broadly oval to round, light green, flat, almost inconspicuous, 0.35–0.45 × 0.30–0.40 mm. ***Stamens***, filaments short, anthers white ovoid, 0.3 × 0.4 mm. ***Fruita*** globose berry, borne on a 1–1.5 mm long, 0.25–0.3 mm diam. narrow-conical transparent pedicel; 0.7–0.8 mm long, 0.6–0.7 mm diam., brown, covered in white 0.05 mm long whitish papillae up to distal third of fruit, style conical, light brown, 0.15–0.2 mm long, 0.20–0.25 mm diam.

##### Distribution and habitat.

Plants have only been found from 1200 to 1500 m of the middle course of the Saña River valley, in the remnants of montane forest, epiphytic or on rocks, always in shaded places. *Peperomiasagasteguii* is endemic to the Saña River valley.

##### Phenology.

Inflorescences appear from October to November; fruits ripen from January to March.

##### Etymology.

This species is dedicated to Abundio Sagástegui (1932–2012), collector of the type, a notable botanist, who worked at both Universidad Nacional de Trujillo and Universidad Privada Antonio Orrego, author of many books and articles, director of the journals “Bulletin of the Society of Botany of the UNT” and *Arnaldoa*, author of four genera and 97 species (Rodríguez and León 2012).

##### Notes.

Most collections were determined initially as *P.trinervis* Ruiz & Pav., a species that seems to be very close, (see Table 4). The main differences are stem color, which is dull dark red and greenish in *P.sagasteguii* compared to the bright red of *P.trinervis*. The indumentum of *P.sagasteguii* seems to be denser and longer, like that of *P.rotundata*. Leaf petioles of *P.trinervis* are shorter and of similar color to its stems. The leaf color of *P.sagasteguii* is bright green adaxially with depressed nerves, while *P.trinervis* has a high color contrast between the dark olive green and the bright silver gray of the nerves. Abaxially leaf color of *P.sagasteguii* is light green, while *P.trinervis* is dark red with light green nerves. *Peperomiasagasteguii* has thicker, more succulent leaves. Finally, spadices of *P.sagasteguii* are much longer. The type collection was determined initially as *P.blanda* (Jacq.) Kunth, which bears no similarity and later it was determined as *P.rotundata* Kunth by R. Callejas, but this sample lacks the constant decussate leaves and inflorescences characteristic of that species. Some branches and leaves can be pseudo-opposite by shortening of the stem, leading to this confusion.

**Table 4.** Comparison of the main differences between *P.sagasteguii* and *P.trinervis*. Data of *P.trinervis* taken from Trelease and Yuncker (1950) and from live cultivated plants.

Features	*P.sagasteguii*	*P.trinervis*
Height (cm)	10–30 (–45)	20–25 or more
Internodes (in cm)	2.5–3 below, 0.8–1 above	2–3 below, 1 above
Stem (in mm)	4–6 diam. at the base, gradually tapering to 1.4–1.6	4–6 diam. at the base, gradually tapering to 1.5–2
Stem (color)	Dull dark red to olive green	Bright dark red
Leaf (petiole in mm)	7–17 long, 1.8–2 wide	3–10 long, 1.2–1.5 wide
Leaf (shape)	Elliptic, elliptic obovate to subrotundate.	Elliptic, elliptic obovate or elliptic obovate.
Leaf (measure)	2.5–4 cm long, 1–2 mm thick, 1.6–2.8 cm wide	2–3.5 cm long, less than 1 mm thick, 1.2–2.5 cm wide.
Leaf (color)	Adaxially bright glossy green, abaxially pale green to pinkish. Young leaves can be pinkish adaxially	Adaxially dark olive green with nerves in gray, abaxially bright dark red, nerves in light green. Young leaves can be purplish
Inflorescence	Terminal in an umbel of 1–3 spadices and sometimes 1–2 individual spadices from upper leaf nodes	Terminal and from the upper nodes.
Rachis of the spadix	4–15 (–20) cm long, 1.5–1.7 mm diam.	10 cm long, 1.8–2 mm diam.

This species belongs to Peperomiasubg.Micropiper (Miq.) Miq. (Frenzke et al. 2015).

##### Additional specimens examined.

**Peru, dept. Cajamarca, prov. Santa Cruz, dist. Catache**: Alrededor de campamento, Bosque Monteseco, borde de trocha sobre troncos secos [Around the campsite, Monteseco Forest, border of track, on dry logs] 1500 m, [06°50'58.6"S, 79°05'53.7"W], 20 Jan 1989, *S. Leyva G. 015* (F 2016269, USM 90066) **Prov. San Miguel, dist. La Florida**: Buenos Aires, La Florida, sobre troncos de Guaba [on trunks of *Inga* sp.] 1000 m, [06°52'27.3"S, 79°07'10"W], 22 Mar 1986, *S. Llatas Quiroz & R. Palacios 1822* (F 1963941). Road from La Florida to Monteseco, just before entering the reserved zone, 1531 m, 6°51'00.7"S, 79°05'58.2"W, 10 Feb 2023, *G. Pino & L.E. Alomía 3831* (USM 333270). **dist. Niepos**: Road from La Florida to Monte Seco (Naranjo), 5.8 km from La Florida, 1395 m, 06°53'25.5"S, 79°06'57.3"W, 1 Mar 2009, *G. Mathieu & L. Symmank 163* (USM); Road from La Florida to Niepos, 1297 m, 06°53'06.9"S, 79°06'47.6"W, 28 Aug 2022, *G. Pino et al. 3641* (USM 333268); Same road, 1393 m, 06°53'25"S, 79°06'57"W, 28 Aug 2022, *G. Pino et al. 3644* (USM 333269).

#### ﻿15. Peperomia symmankii

Taxon classificationPlantaePiperalesPiperaceae

Pino & Samain sp. nov.

999CDA52-689F-5B5E-9676-0FD6AA0B54D4

urn:lsid:ipni.org:names:77317655-1

Fig. 10A–F

##### Type.

**Peru**, dept. Cajamarca, prov. San Miguel, dist. La Florida: road from Cayaltí to La Florida, 731 m, 06°51'57.6"S, 79°08'32.3"W, 1 Mar 2009, *G. Mathieu & L. Symmank 162* (holotype: USM[255772]!).

**Figure 10.**
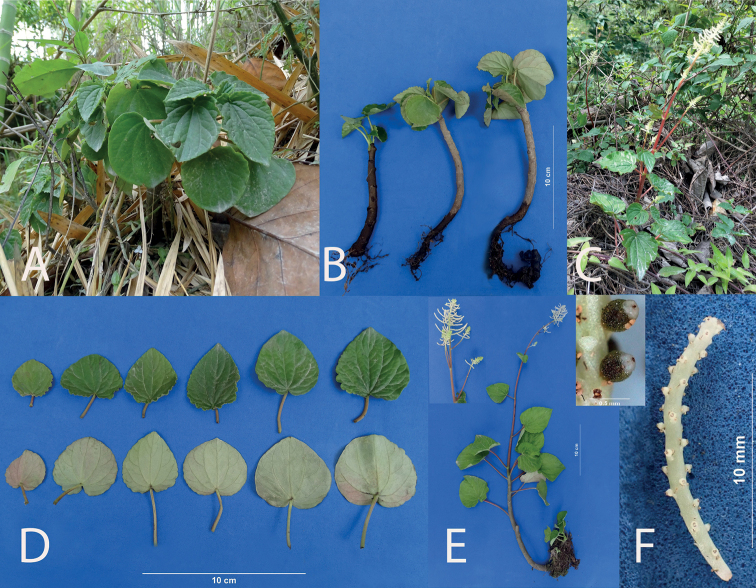
*Peperomiasymmankii* Pino & Samain, sp. nov. **A** plant in habitat **B** plants *ex situ* in several stages of development **C** plant in habitat in blossom **D** detail of the leaves **E** plant ex situ in anthesis with bracts. (Detail: mature panicle) **F** young spadix (Detail: Mature spadix with fruits).

##### Diagnosis.

Perennial, succulent, caulescent, terrestrial herb, similar to *P.ricardofernandezii* Pino & Samain, but shorter, less branched, and with more tortuous stems. Leaves are smaller (2.5–4.5 cm long compared to 4.5–8 cm long), more widely ovate, with an obtuse or rounded apex instead of acute-acuminate. Petioles are shorter (0.3–3.5 cm long compared to 2–4 cm), lighter in color, and attached closer to the cordate base, which is sometimes auriculate and overlapping compared to the truly peltate insertion of *P.ricardofernandezii*. The inflorescence is narrower and longer but otherwise very similar, with longer spadices (3–20 mm compared to 2–4 mm).

##### Description.

Perennial, succulent, caulescent, terrestrial ***herb***, 12–20 cm when vegetative, up to 5 cm when flowering, growing on soil among rocks at slightly shaded places, near water courses. Vegetative ***stem***, 0.6–1.5 cm diam. At the base, light gray brownish, erect at first, then sometimes decumbent, rooting from the base, terete in the rainy period, slightly furrowed under dry conditions, with flat or projected spirally arranged round to reniform scars, 0.15–0.3 cm tall, 0.25–0.45 cm wide, distanced every 0.5–1 cm, corresponding to decurrent petioles. ***Leaves*** in young plants perennial, alternate, spirally attached, present at the distal 2–4 cm, glabrous; petiole reniform in cross-section, 0.5–3.5 cm long, 2–2.5 mm wide, erect or oblique upwards, light green or with a reddish blush, inserted almost at the base of the leaves, subpeltate at 1–2 mm from abaxial base; lamina widely ovate, 2–4.5 cm long, 2–4.5 cm wide at proximal third, 2.5–5 cm wide at the middle, 2.5–4 cm wide at distal third, apex obtuse, occasionally rounded, base cordate, sometimes auriculate and overlapping; adaxially dull green, flat to concave. 7-palmatinerved, nerves depressed; margin entire, undulated at distal half; abaxially very light green, sometimes reddish, nerves slightly carinate, darker green. A reproductive stem develops in the rainy season, from the apex the first time or from the next most distal node, with bracts similar to leaves, their lamina is first corrugate with undulate margins, and reddish abaxially, later they become larger, 4–7 cm long, 5–7 cm wide at proximal third, 3–6.5 cm wide at the middle, 4–5.5 cm wide at distal third, flat, adaxially bright green, abaxially very light green with nerves less conspicuous, apex acute to obtuse, base cordate, margins entire, petiole 4–7 cm long, light green. All bracts and inflorescences are deciduous after flowering. ***Inflorescence*** 2–5 (–10) panicles born alternately from the distal stem towards the apex, appearing from February to March. Each panicle is a raceme of 15–45 conferted, spirally arranged, horizontally inserted spadices around a vertical central axis, gradually opening from base to apex, longer and whiter at the base and shorter and greener distally. Central axis 10–30 cm long in total, 0.4–0.5 mm diam. at the base, gradually tapering to 0.9–1 mm, terete, light green to reddish, whitish towards the apex; ***peduncle*** terete or slightly funnel-shaped distally, 1–2 mm long, 0.3–0.4 mm diam., bright white; ***rachis*** 3–20 mm long, 0.4–0.5 mm diam., longitudinally furrowed, bright white. ***Floral bracts*** peltate, discoid, greenish white, 0.25–0.3 mm diam. ***Stamens*** white, pink when dry, filaments 0.2 mm long, transparent, anthers white ovoid, 0.15–0.20 mm. ***Ovary*** globose, white, 0.35–0.45 mm diam., stigma fimbriate. ***Fruit*** an ovoid berry, 0.75–0.85 mm long, 0.55–0.65 mm diam., olive green-brownish, minutely papillate, pedicel inconspicuous, style very broadly conical, white, stigma brown.

##### Distribution and habitat.

Plants are rare and have been found only at four spots from 700 to 2400, semi-shaded on soil accumulated among huge rocks, on moist places near watercourses.

##### Phenology.

Inflorescences appear from December to March; fruits ripen from February to April.

##### Etymology.

The epithet is dedicated to Lars Symmank, a botanist from Dresden, Germany, who together with Guido Mathieu collected the specimen of this new species in the first expedition to Peru organized by Ghent University in 2009. He worked in several studies of subgenus Tildenia (Mathieu et al. 2011; Samain et al. 2011; Symmank et al. 2011).

##### Notes.

The closest species to *P.symmankii* is *P.ricardofernandezii* from Piura. This species is mainly terrestrial, taller, and many branched with a bush-like appearance contrasted to the creeping habit of *P.symmankii*. The main difference is the leaf size and shape: *P.symmankii* has relatively smaller leaves, more widely ovate, with an obtuse to round apex, compared to the narrow ovate, acute-acuminate leaves of *P.ricardofernandezii*. Petioles of the new species are shorter, lighter in color, attached closer to the leaf base, which is truly cordate, even sometimes auriculate and overlapping, compared to the more peltate insertion in *P.ricardofernandezii*. The inflorescence of *P.symmankii* is narrower and longer but otherwise very similar, with slightly longer spadices (Table 5).

**Table 5.** Comparison of the main differences between *P.symmankii* and *P.ricardofernandezii*.

Features	*Peperomiasymmankii*	*Peperomiaricardofernandezii*
Height (vegetative in cm)	12–20	10–50
Height (flowering, in cm)	25–45	15–60 or more
Petioles (surface and color)	Glabrous, light green or with reddish blush	Glabrous, burgundy red.
Petioles (length and insertion)	0.5–3.5 cm	2–4 cm
	Subpeltate 1–2 mm from abaxial base	Subpeltate 2–3 mm from abaxial base
Leaves (shape and color)	Widely ovate, apex acute, base cordate; adaxially dull green, abaxially very light green, sometimes with a slight blush.	Narrowly ovate, apex acute to subacuminate, base subcordate; adaxially light green, abaxially reddish to burgundy red.
Leaves (size)	2–4.5 cm long	4.5–8 cm long
	2.5–4 cm wide at distal third	1.5–3 cm wide at distal third
	2.5–5 cm wide at the middle	2.5–6 cm wide at the middle
	2–4.5 cm wide at proximal third	2.5–5.5 cm wide at proximal third
Vegetative stem	Perennial, usually one branch from subterminal bud.	Perennial, 1–3 branches from subterminal bud.
Bracts (petiole)	4–5 cm long, light green, inserted subpeltate at 1–3 mm from abaxial base	2–10 cm long, red, inserted subpeltate at 2–5 mm from abaxial base
Bracts (shape and size)	Widely ovate	Ovate
	4–7 cm long	4.5–8 cm long, 2.5–6 cm wide,
	3–7 cm wide,	apex acuminate and clearly acute distally
	apex acute to obtuse	
Central axis of panicle (length in cm)	10–30	3.5–7
Rachis of spadix (length in mm)	3–20	2–4

This species belongs to Peperomiasubg.Panicularia Miq. (Frenzke et al. 2015).

##### Additional specimens examined.

**Peru, dept. Cajamarca, prov. San Miguel, dist. La Florida**: El Papayo bridge over Saña River, East bank, 10 m to North, under the shade of *Guaduaangustifolia* plants, on rocks, 731 m, 06°51'57.6"S, 79°08'32.3"W, 28 Aug 2022, *G. Pino et al. 3639* (USM 333272). El Papayo, sobre materia orgánica de las rocas. [El Papayo, on rocks with the decayed organic matter] 850 m, [06°51'S, 79°08'W], 1 Feb 1986, *S. Llatas Q. 1770* (HUT 22496); Road from Florida to Monteseco, 1095 m, 6°51'41.3"S, 79°07'08.9"W, 10 Feb 2023, *G. Pino & L.E. Alomía 3832*; (USM 333273). **Dist. Bolívar**: Bosque Oscuraná, Caserío el Nogal, Bosque Húmedo [Oscuraná Wet Forest, El Nogal Hamlet], 2400 m, [6°56'51.0"S, 79°08'04.0"W], Sep 9 2003, *A. Juárez et al. s.n.* (HLL 435, HUT 42219). **Prov. Santa Cruz, dist. Catache**: Carretera al Bosque de Monteseco, borde de Carretera. [Road to Monteseco forest, border of road], 1200 m, [06°51'21"S, 79°06'42"W], 20 Jan 1996, *E. Rodríguez 750* (HUT 30979).

#### ﻿16. Peperomia vivipara

Taxon classificationPlantaePiperalesPiperaceae

Pino, Samain & L.E. Alomía sp. nov.

D93406AC-CCD3-521F-AD44-4210FDA4010F

urn:lsid:ipni.org:names:77317656-1

Fig. 11A–F

##### Type.

**Peru**, dept. Cajamarca, prov. Santa Cruz, dist. Catache: Monteseco Forest. 1500 m, [06°50'58.6"S, 79°05'53.7"W], 19 Dec 1984, *A. Sagástegui et al. 12396* (holotype: HUT [19867] !; isotype: F [2038843] !).

**Figure 11.**
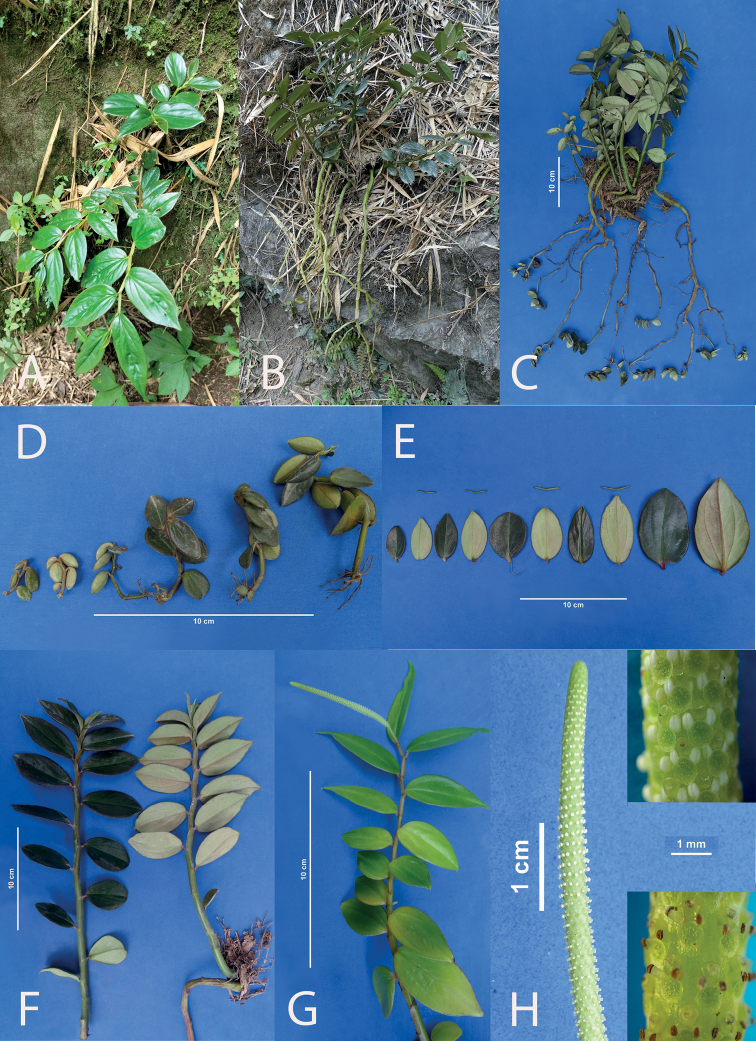
*Peperomiavivipara* Pino, Samain & L.E. Alomía, sp. nov. **A** young plant in habitat **B** mature plant in habitat showing offsets **C** plant *ex situ* showing offsets **D** propagules of different sizes just detached from mother plant **E** detail of leaves **F** detail of a branch: adaxial side (left), abaxial side (right) **G** branch with terminal spadix **H** spadix in anthesis.(Detail: Above, before; and below, after development of stamens).

##### Diagnosis.

Perennial, semi-succulent, caespitose herb similar to *P.alata*, but more succulent, with thicker and more stout branches, leaves are shorter, elliptic, acute based, instead of lanceolate and long acuminate. Leaf bases are obtuse or subtruncate instead of cuneate. After flowering, propagules are developed at the apex of old branches that readily wither to assure vegetative propagation.

##### Description.

Perennial, semi-succulent, caespitose ***herb***, growing terrestrial, epiphytic, or saxicolous in the shade of other plants, 30–45 cm tall. Roots fibrous, emerging from the base of plants, 0.7–1 mm diam., light brown. ***Stem*** stoloniferous at the base, from which 3–10 deciduous ***branches*** emerge, straight erect, sometimes in zig-zag towards the apex, terete at the base, then prominently winged towards the apex with a triangular section, dark olive green, slightly puberulous at the base, then glabrescent towards the apex, 0.7–1 cm diam. At the base, gradually tapering to 0.3–0.5 mm at the apex, rarely branched close to the base, internodes 2.5–5 cm at the base gradually descending to 0.5–0.7 cm towards the apex, wings prominent at the distal half of the stems, from below the node towards the node after the next, semitransparent, 1–2 mm tall. ***Leaves*** alternate, distichous, glabrous, elliptic to elliptic ovate; petiole short, decurrent to next opposite node, reniform in section, channeled above, reddish green-pinkish, 0.2–0.4 (–1) cm long, 1.5–2 mm wide; lamina 3–6.5 cm long, 0.5–1.5 mm thick, 1.8–3.2 cm wide, apex acute to very slightly acuminate, slightly recurvate, base sub truncate; adaxially bright glossy green in young leaves, then very dark army green and even bluish, flat to slightly canaliculate, 3–5-palmatinerved, nerves depressed; margin entire; abaxially very light green with a pink blush, 3-palmatinerved, nerves elevated. ***Inflorescence*** simple terminal or up to 3 individually from upper leaf nodes opposite to the leaves; ***peduncle*** terete to infundibuliform, bright green, 2–9 mm long, 1.4–1.5 mm diam.; ***rachis*** 3–6 cm long, 2–3 mm diam., light green. ***Floral bracts*** rotundate, light green, flat, almost inconspicuous, only darker green than rachis, 0.4–0.5 mm diam. ***Stamens***, filaments transparent, 0.2 mm diam., 1.5–3 mm long, anthers white ovoid, 0.4–0.5 mm long, 0.3–0.4 mm wide. ***Fruit*** not seen. ***Propagule*** developing at the apex of the branch, 1–5 cm tall, persistent until the whole stem dries.

##### Distribution and habitat.

Plants grow from 1500 to 1800 m of the middle course of the Saña River valley, in the remnants of montane forest, epiphytic or on rocks, always in shaded places.

##### Phenology.

Inflorescences appear from December to March; fruits ripen from February to April.

##### Etymology.

The epithet stands for the most striking feature of this plant, the production of offsets directly from the mother plant, bypassing germination, as a method of survival in extreme weather conditions, from the Latin *viviparus* (that brings forth its young alive).

##### Notes.

The closest species to *P.vivipara* is *P.alata* Ruiz & Pav., a species of wide distribution in Mexico, Central America, and all countries of South America except Chile, Argentina, and Uruguay, in tropical forests at lower elevations. Plants are similar in habit and size, they share the zig-zag winged internodes, although *P.vivipara* is stouter, with thicker and more erect branches with longer internodes. The main difference is in leaves, which are shorter, elliptic with an acute short apex and an obtuse or almost truncate base in *P.vivipara* compared to the lanceolate, long acuminate, and acute-based leaves of *P.alata*. A further difference is that *P.alata* does not produce propagules at the distal stems. (Table 6) These plantlets, that drop readily and root close to the mother plant propagating the plants vegetatively, have not been seen in any other species of *Peperomia* and give the name to the epithet of the species.

**Table 6.** Comparison of the main differences between *P.vivipara* and *P.alata*.

Features	*Peperomiavivipara*	*Peperomiaalata*
Height	30–45 cm	30 cm or more
Stem (diam. at base)	7–10 mm	3–8 mm
Internodes (distance)	2.5–5 cm	1–3 cm
Petioles (length and diam.)	2–10 mm long	3–20 mm long
	1.5–2 mm diam.	0.8–2 mm diam.
Leaves (shape)	Elliptic to elliptic ovate,	Elliptic to elliptic-lanceolate,
	apex acute to slightly acuminate,	apex long acuminate,
	base obtuse, subtruncate	base acute, cuneate
Vegetative leaves (size)	3–6.5 cm long	4.5–15 cm long
	1.8–3.2 cm wide	2–4.5 cm wide
Spadices (length)	3–6 cm	6–8 (–15) cm
Propagules at distal stem	Present	Absent

This species belongs to Peperomiasubg.Micropiper (Miq.) Miq. (Frenzke et al. 2015).

##### Specimens examined.

**Peru, dept. Cajamarca, prov. Santa Cruz, dist. Catache**: Monteseco, 5 km above, on the path to Chorro Blanco, 1500 m, [06°50'58.6"S, 79°05'53.7"W], 16 Mar 1986, *M.O. Dillon & A. Sagástegui 4366* (F 1993688); Same place, on the path below the campsite, 1450 m, [06°51'10.8"S, 79°06'09.4"W], 19 Mar 1986, *M.O. Dillon & A. Sagástegui 4432* (F 1993348); Monteseco, 3 km NE, 1750 m, 06°50'51.6"S, 79°06'14.7"W, 13 May 1987, *J. Santisteban C. & J. Guevara B. 044* (HUT 24730, F1995346). **Prov. San Miguel, dist. Niepos**: Road from La Florida to Monte Seco (Naranjo), 14.5 km from La Florida, 1643 m, 06°54'04.8"S, 79°07'33.5"W, 1 Mar 2009, *G . Mathieu & L. Symmank 165* (USM 258839); Road from Naranjo to La Florida, 1632 m, 06°54'00.2"S, 79°07'59.0"W, Jul 26 2020, *G. Pino & L.E. Alomía 3214*; Same road, 1613 m, 06°54'03.3"S, 79°07'33.5"W, 26 Jul 2020, *G. Pino & L.E. Alomía 3215* (USM 330866); Idem, 1612 m, 06°54'03.3"S, 79°07'33.5"W, Feb 2 2023, *G. Pino & L.E. Alomía 3833* (USM 333271); Idem, 1393 m, 06°53'25.3"S, 79°06'57.7"W, 26 Jul 2020, *G. Pino & L.E. Alomía 3216* (USM 330867); Road from La Florida to Niepos, 1393 m, 06°53'25"S, 79°06'57"W, 28 Aug 2022, *G. Pino et al. 3643* (USM 330886); Same road, 1613 m, 06°54'03.3"S, 79°07'33.5"W, 28 Aug 2022, *G. Pino et al. 3645* (USM 330887).

## ﻿Discussion

This research found 16 taxa of *Peperomia* in a radius of less than 25 km (Fig. 2). Previous studies had reported nine species of *Peperomia* in the protected area of Udima, north of the town of Monteseco (Sagástegui and Dillon 1991) and only four in the still unprotected forest La Oscurana in Bolívar (Juárez et al. 2005). It is noticeable that previous studies focus on “relict forests” that represent a sample of the former extension of the natural extents of these woodlands, but neglect to sample semi-arid environments that can also be rich in species, as we show in this article. This species richness could be explained by the influence of the AHZ, but the closest valleys to the north and south of Río Saña, also inside this zone have very scarce collections of this genus. Perhaps they deserve more research to verify if they share the same biodiversity.

We have observed that the highest diversity of *Peperomia* species occurs between 500 and 1600 m, although more rainfall and vegetation can be expected at higher altitudes. The species found in the upper layers (*P.hispidula*, *P.rotundata*. *P.microphylla*, *P.galioides*) have a broad distribution in the department of Cajamarca (Pino 2004) and even in the rest of the Americas. The fact that the endemic or rare species are concentrated in the middle range of mountain systems is an observation consistent with other floral studies (Vergara et al. 2017) but has no demonstrated explanation yet.

Another observation is that in the lower range, where more droughts threaten the diversity of species of *Peperomia*, we have found two species of subgenus Fenestratae and one of subgenus Panicularia. These subgenera are restricted to Ecuador and Peru, and they show an extreme adaptation to prolonged periods of drought through succulence. Other families adapted to this desertic environment, such as Cactaceae, – are also rich in diversity in this lower layer (Mischler 1988; Alomía 2020). Finally, our findings support the hypothesis that there are still many species of *Peperomia* in Peru awaiting their discovery.

## Supplementary Material

XML Treatment for Peperomia cacaophila

XML Treatment for Peperomia cymbifolia

XML Treatment for Peperomia cymbifolia Pino var. occidentalis

XML Treatment for Peperomia dolabriformis

XML Treatment for Peperomia emarginulata

XML Treatment for Peperomia fraseri

XML Treatment for Peperomia galioides

XML Treatment for Peperomia haematolepis

XML Treatment for Peperomia hispidula

XML Treatment for Peperomia inaequalifolia

XML Treatment for Peperomia microphylla

XML Treatment for Peperomia pilocarpa

XML Treatment for Peperomia riosaniensis

XML Treatment for Peperomia rotundata

XML Treatment for Peperomia sagasteguii

XML Treatment for Peperomia symmankii

XML Treatment for Peperomia vivipara

## Supplementary materials

﻿Supplementary material 1 Localities of *Peperomia* species from the Saña River Valley, Peru Data type COL (excel document) Explanation note Database of localities of *Peperomia* species found at the Saña River Valley Peru, data obtained from Herbarium sheets, personal collections, or calculations based on data from Herbarium labels. https://binary.pensoft.net/file/839983This dataset is made available under the Open Database License (http://opendatacommons.org/licenses/odbl/1.0/). The Open Database License (ODbL) is a license agreement intended to allow users to freely share, modify, and use this Dataset while maintaining this same freedom for others, provided that the original source and author(s) are credited.Guillermo Eloy Pino Infante, Marie-Stéphanie Samain, Luis Enrique Aarón Alomía Collazos
